# 3D Modeling Study of Bubble-Driven Flow and Its Interaction with Cell Operation

**DOI:** 10.1021/acsomega.5c09258

**Published:** 2025-11-06

**Authors:** Samuel Théberge, Lukas Dion, Lászlo Kiss, Thomas Roger, Simon-Olivier Tremblay, Sébastien Guérard, Jean-François Bilodeau

**Affiliations:** † Department of Applied Sciences, 14661 University of Quebec in Chicoutimi, 555 Bd de l’Universite, G7H 2B1 Chicoutimi, Canada; ‡ Rio Tinto, Arvida Research and Development Center, 1955 Bd Mellon, G7S 2H8 Jonquière, Canada

## Abstract

A detailed three-dimensional model of carbon dioxide generation and movement beneath the anode in an aluminum electrolysis cell has been developed. By incorporation of localized current density and multiple nucleation sites, the model captures the transient behavior of the anode–cathode distance (ACD) and the deformation of the bath–metal interface (BMI) caused by bubble dynamics. It also evaluates the pot’s response in terms of turbulent kinetic energy, providing insights into alumina dissolution efficiency and heat transfer mechanisms. The model further investigates how the MHD-induced flow direction and evacuation channel geometries impact bubble behavior, voltage fluctuations, and thermal distribution. The results not only align with existing experimental data but also shed light on subtle interplays within the cell environment. Notably, the orientation of the MHD flow emerges as a decisive factor in the local bubble overvoltage and the heterogeneity of alumina mixing. This study provides industry with actionable insights into optimizing cell design parameters by taking into consideration the impact caused by their operational ACD range, the evacuation channel width, and the relative MHD flow direction.

## Graphical abstract

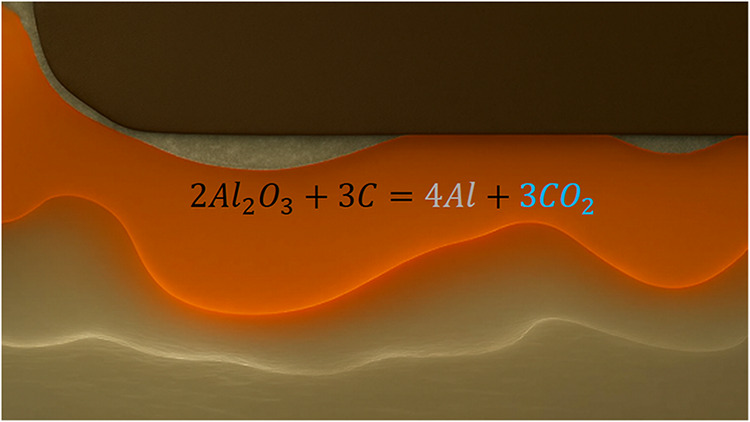

## 1 Introduction

### 1.1 Background

Most aluminum smelters use the Hall-Héroult process to produce aluminum through electrolysis of alumina (Al_2_O_3_). In this process, molten cryolite is used as a solvent to dissolve powdered alumina at temperatures around 960 °C, well below its melting point of 2072 °C.^1,2^ Figure 1 shows a typical arrangement of a pot by using this process.

**Figure 1:**
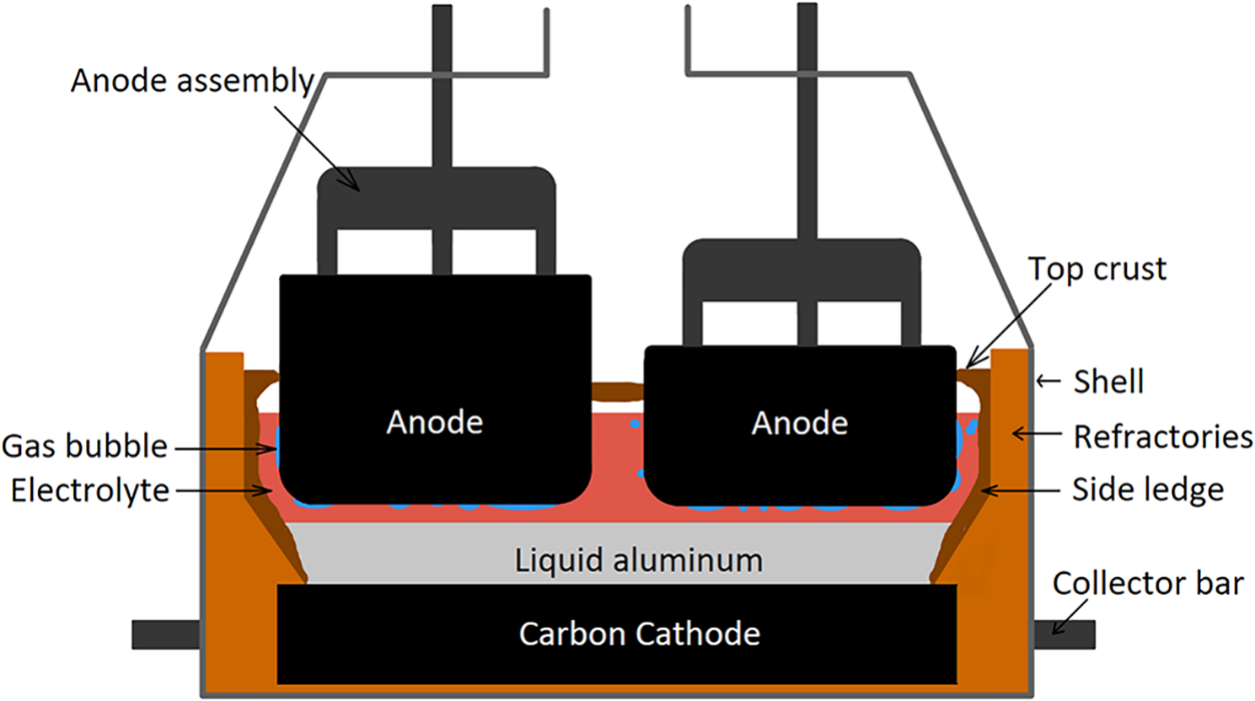
Components of the aluminum electrolysis pot.

Aluminum is generated by the electrolysis of alumina according to the following simplified equation:

1 $$2{Al}_{2}O_{3}+3C=4Al+3{CO}_{2}E0=-1.18\;V$$

The aluminum produced accumulates at the bottom of the cell with liquid aluminum already present. On the other side, the oxygen in the alumina reacts with the carbon of the anodes to produce gaseous CO_2_.

As mentioned, eq 1 is the simplified global reaction. In reality, a number of intermediate reactions take place, and they can influence the correct operation of the pot. In particular, other gases can be produced. Among others, CO is created according to the Boudouard equation (eq 2), HF is generated due to humidity (eqs 3 and 4) as well as the residual water content trapped in the alumina, and perfluorocarbons (PFCs) are generated in higher volume when the alumina concentration in the electrolyte becomes too low (eqs 5 and 6).^1–3^

2 $${\mathrm{CO}}_{2}+C=2\mathrm{CO}$$

3 $$2\mathrm{Al}F_{3}+{3H}_{2}O=6\mathrm{HF}+{\mathrm{Al}}_{2}O_{3}$$

4 $$3\mathrm{NaAl}F_{4}+{3H}_{2}O=6\mathrm{HF}+{\mathrm{Al}}_{2}O_{3}+{\mathrm{Na}}_{3}{\mathrm{AlF}}_{6}$$

5 $$4{\mathrm{Na}}_{3}{\mathrm{AlF}}_{6}+3C\to 4\mathrm{Al}+3{\mathrm{CF}}_{4}+12\mathrm{NaF}$$

6 $$2{\mathrm{Na}}_{3}{\mathrm{AlF}}_{6}+2C\to 2\mathrm{Al}+C_{2}F_{6}+6\mathrm{NaF}$$

However, in normal operation, the gas produced under the anodes with the greatest mass is CO_2_ and was selected as the only gas represented in the model. However, it is essential to note that when produced in significant quantities, other gases can behave differently from CO_2_. For example, PFCs (CF_4_ and C_2_F_6_) have higher wettability and tend to adhere more strongly to the anodes. This is precisely what happens when anode effects produce large quantities of PFCs, resulting in greater insulation of the underside of the anode by these gases.

### 1.2 Improving Faraday Performance and Energy Efficiency

To maximize metal production while limiting the generation of greenhouse gases, aluminum producers are constantly looking for ways to optimize their energy and current efficiencies. In particular, the **current efficiency** corresponds to the amount of aluminum that can theoretically be produced for a given electric charge, relative to the actual amount obtained. **Energy efficiency**, on the other hand, is the amount of energy theoretically required to produce aluminum compared with the amount actually used.

A better understanding of the phenomenon of bubble production and flow can help improve the efficiencies, particularly by enhancing the stability of the bath–metal interface, alumina dissolution, and thermal equilibrium. Indeed, the bubbles that escape create movement at the bath–metal interface (BMI). This wavy movement of the metal can lead to the proximity of aluminum and CO_2_ bubbles, promoting the reverse reaction to electrolysis (eq 7).

7 $$2\mathrm{Al}+3{\mathrm{CO}}_{2}={\mathrm{Al}}_{2}O_{3}+3\mathrm{CO}$$

Also, by limiting the bubble coverage beneath the anode, it is possible to decrease the resistance to current flow, thereby reducing the required electrical power. Similarly, by efficiently redirecting the bubbles, the desired heat transfer to the sidewalls can be obtained for better control of the thermal balance of the pot.

Finally, proper dissolution and distribution of alumina can help reduce the occurrence of anodic effects in the cell. Anodic effects happen when the alumina concentration becomes too low to sustain the passage of the electric charges. At this point, the electrolysis of cryolite becomes necessary for the process, and perfluorocarbons (PFCs) are produced as a result of these new chemical reactions. These effects are highly detrimental (chemically, thermally, and electrically) to the electrolysis process and must therefore be avoided as much as possible.

## 2 Literature Review

Authors have produced numerical models of fluid flow in the electrolysis cell with the aim of improving the understanding of the process. However, since the process is very complex and the models can become heavy in terms of calculation, the authors produced their models in a precise way with the aim of observing targeted phenomena. Among all these authors, it is possible to retain some interesting approaches to the creation of a new model in order to accurately represent the project’s focus.

As part of their research, Einarsrud et al.^4^ developed a detailed three-dimensional model of an aluminum electrolysis cell. For this purpose, the authors used the ANSYS FLUENT software. This transient model represents the fluid flow, the current distribution, the magnetohydrodynamics (MHD), and the alumina distribution in the bath. A number of objectives were targeted by this model: to determine the average deformation of the metal pad due to MHD, to determine the frequency of bubble escape, and to gain a better understanding of fluid movement in the cell and therefore of alumina transport at the same time. First, the results showed that the metal pad deforms mainly along the length of the cell with the highest point at its center. The height at the extremities can be as much as 25% lower than that at the highest point. Similarly, this model shows that metal flow velocity can reach up to 30 cm/s but has an average velocity of around 10 cm/s. They were also able to observe the importance of the anode angle on the accumulation of bubbles beneath it. Increasing the angle significantly reduces the coverage of bubbles.

Zhan et al.^5^ have produced two three-dimensional models in the transient domain: one representing an entire cell; the other considered only three anodes. The models have two phases, namely, the CO_2_ and the electrolytic bath. These models provide velocity fields and visualize bubble flow. In particular, their study showed that larger bubbles tend to stay on the edges of the anode, where smaller bubbles are toward the center. This demonstrates the bubbles’ resistance to leaving the underside of the anode. Although Einarsrud et al.^4^ reported average velocities of around 10 cm/s, Zhan et al. presented average velocities of around 3 cm/s. This difference may be due in part to the fact that Einarsrud’s model represents the effect of MHD, while Zhan’s does not.

Feng et al.^6^ also reproduced three anodes to observe bubble flow in the transient regime. ANSYS CFX was used for this study. The effect of slots on the flow was also studied. Among other things, the water velocities, which represent the cryolite, are extracted and range from −15 to 15 cm/s. In their simulations with a slot, they found in some cases a higher proportion of bubbles escaping toward the central channel, in others toward the side channel, and sometimes equal between the two. In their case, this behavior appeared to be random rather than parameter controlled.

Severo et al.^7^ used ANSYS CFX to produce a model representing a single anode and another model representing the entire cell. The authors used an MHD flow that had been determined in a previously made model. The main aim of this work was to determine the impact of different anode slot configurations in order to improve the cell performance. The model with a single anode shows, among other things, the importance of slots to limit the volume of gas trapped under the anode. This is particularly true for the half-submerged longitudinal slot, which evacuates the bubbles better, with a 47.6% reduction compared with the case without the slot. In the same spirit, it shows the significant effect of MHD on bubble evacuation, with a reduction of 22% in bubble coverage.

Finally, Gusberti and Severo^8^ once again used ANSYS CFX to study the effect of different anode geometries on bubble behavior. Among other things, they studied the effect of the anode inclination and slot configuration. More specifically, the authors sought to better understand the effect of these parameters on the voltage drop created by the bubbles with a view to reducing it. Their model also shows that adding slots reduces the tension. The first slot reduced the voltage by 89 mV, while the second and third slots reduced it by just 17 mV, and then by a further 10 mV. Finally, no effect was observed when the slot immersion was modified or when the slot was blocked on the side wall direction.

### 2.1 Summary of Literature and Potential Impact on This Work

Various parameters must be determined to create a model such as that described above. In this context, some of the key parameters used in these models are summarized below, with the aim of drawing conclusions about the relevance of using one rather than another.

The k-epsilon turbulence model is well suited to low-speed, moderately turbulent models.^9^ In fact, all authors discussed in the literature review^4–8^ have used this turbulence model in their work since it offers a good compromise between robustness and computing time.

In addition, in order to make an effective comparison between models, it is necessary to know whether the third phase (liquid aluminum) is represented and whether the effect of MHD is modeled or not. Then, the interface tracing method is particularly important to consider, as it greatly influences the results. Table 1 gives this information for all of the papers discussed in the literature review.

**Table 1:** Summary of the Authors’ Modeling Parameters

**author**	**presence of liquid aluminum**	**representation of the MHD**	**interface tracking method**
Einarsrud et al.^4^	yes	yes	volume of fluid
Zhan et al.^5^	no	no	Euler–Euler
Feng et al.^6^	no	no	Euler–Euler
Severo et al.^7^	no	yes	volume of fluid
Gusberti and Severo^8^	no	no	not mentioned

Table 1 shows that most authors avoided reproducing the liquid metal to simplify the models. In doing so, the effect of aluminum on cell equilibrium and BMI deformation is neglected. One of the aims of this work is to fill this gap.

Furthermore, in each of these models, it is interesting to observe the boundary conditions used by the authors. This exercise was carried out as part of our paper presented at the 41st International ICSOBA Conference,^10^ and Table 2 presents a similar summary of the boundary conditions used by the various authors.

**Table 2:** Summary of the Boundary Conditions Used by the Authors^a^

	fluidic boundary conditions	electrical boundary conditions	
**authors**	**under the anode**	**anode**	**cathode**
Einarsrud et al.^4^	user-defined function (UDF)	0 V	imposed current density
Zhan et al.^5^	mass inlet	not applicable	not applicable
Feng et al.^6^	mass inlet	not applicable	not applicable
Severo et al.^7^	mass inlet	not applicable	not applicable
Gusberti and Severo^8^	uniform generation	imposed voltage	imposed current density
**present work**	user-defined function (UDF)	0 V	imposed current density

a Source: Adapted with written permission from ICSOBA 2023, copyright 2023, UQAC.

Of these five models, Einarsrud’s^4^ and Gusberti’s^8^ are the closest to what is aimed for in this work. Moreover, the method they used to determine the electrical conditions at the anode and cathode best represents the reality of a cell. Current density is imposed at the cathode, while constant voltage is imposed at the anode. This way, if a bubble is present at a precise location under the anode, the current density will be reduced locally since it is electrically isolated by the gas.

As far as bubble generation is concerned, both authors used a function that generates bubbles everywhere under the anode rather than at a single targeted point. In the case of Einarsrud, the method used is not precisely explained. In the case of Gusberti, the generation is uniform under the anode.

Although the three other models have different objectives than the model presented in this work, they demonstrate the option of generating bubbles in a more simplified way with a mass inlet.

## 3 Modeling Method

This work follows an initial stage in which a two-dimensional model was produced in order to calibrate the model and determine which parameters were the most important to consider for a three-dimensional model. The previous work was presented at the 41st International ICSOBA Conference.^10^

### 3.1 Modeling Parameters Used

Numerical modeling was carried out with the commercial software ANSYS FLUENT, using the Volume of Fluids (VOF) method coupled with the Level Set function. The VOF method is efficient for modeling immiscible fluids, as it is the case for the fluids present in the cell. Moreover, the Level Set function allows better management of the boundary between the phases and surface tensions, giving a more realistic representation of CO_2_ bubbles in the electrolyte.

The standard k-ε turbulence model is used since it offers a good compromise between simulation accuracy and computation time. Indeed, although this model may be less accurate than some other models with more than two equations, the aim of this work is to carry out a parametric study, i.e., to be able to simulate various cases in order to compare behavior between them. On another note, as shown in the literature review, most authors who produce this type of model choose the k-e turbulence model, showing its relevance.

In the simulations, the three main phases in a cell are represented: carbon dioxide, electrolyte, and liquid aluminum. Despite the increase in simulation time, the choice was made to add liquid aluminum to obtain a more realistic current distribution, which leads to a physically accurate current distribution following the deformation of the BMI. Subsequently, the current distribution has an impact on preferential bubble generation zones, which are managed by the UDF which also enhance the accuracy of bubbles generation. For this reason, the electrical module is activated in the model.

Finally, it is important to note that MHD forces are not represented in the model. An important part of the vertical deformation of the bath–metal interface is missing in the simulations. On the other hand, velocities in the horizontal plane are imposed, based on previously realized external models to represent this effect on the motion of the electrolytic bath and bubbles.

### 3.2 Equations

The VOF model is used to simulate nonmiscible fluids and trace their interface. So, since various phases may be present in a cell, the model must determine the fraction of each phase in the cells..^9^

α_*q*_ = 0: The cell does not contain *q*th fluid

α_*q*_ = 1: The cell is full of *q*th fluid

Thus, a cell with 50% of phase 1 and 50% of phase 2 will have an α_1_ of 0.5, an α_2_ of 0.5, and an α_3_ of 0 since this model represents three phases.

#### 3.2.1 Materials Properties

The material properties are defined as a weighted average from all of the fluids involved in the cell. For example, eq 8 defines the density in a cell.

8 $$\rho =\sum \alpha_{q}\rho_{q}$$

ρ_*q*_ is the density of the *q*th fluid

α_*q*_ is the volume fraction of the *q*th fluid in the cell

ρ is the density in the cell

The properties calculated in this way include density, electrical conductivity, and viscosity.

#### 3.2.2 Volume Fraction Equation

The volume fraction equation tracks the interface between the phases.

9 $$\frac{1}{\rho_{q}}\left[\frac{\partial }{\partial t}\left(\alpha_{q}\rho_{q}\right)+\nabla \cdot \left(\alpha_{q}\rho_{q}{\mathrm{v⃗}}_{q}\right)\right]=S_{\alpha_{q}}+\sum_{p=1}^{n}\left({ṁ}_{\mathrm{pq}}-{ṁ}_{\mathrm{qp}}\right)$$

where *ṁ*_*pq*_ *and ṁ*_*qp*_ are respectively the mass transfer from phase p to q and the mass transfer from phase *q* to *p*; *v⃗*_*q*_ corresponds to the velocity of the fluid in the *q*th phase; and *S*_α_*q*__ is a source term. In this model, the value of the source term is governed by a user-defined function that manages CO_2_ generation under the anode.

#### 3.2.3 Momentum Equation

The Level Set function tracks the interface between fluids for a better-defined representation than that with VOF alone. The Level Set function φ­(*x*,*t*) is defined such that φ­(*x*,*t*) = 0 corresponds to the interface between two fluids.

10 $$\;\varphi \left(x,t\right)=\{\begin{array}{l} +\left|d\right|\;\mathrm{if}\;x\;\mathrm{is}\;\mathrm{on}\;\mathrm{the}\;\mathrm{primary}\;\mathrm{phase}\\ 0\\ -\left|d\right|\;\mathrm{if}\;x\;\mathrm{is}\;\mathrm{on}\;\mathrm{the}\;\mathrm{secondary}\;\mathrm{phase} \end{array}$$

where *d* represents the signed distance from the interface with positive values in the primary phase and negative values in the secondary phase.

Finally, the **momentum equation** is given by

11 $$\frac{\partial \left(\rho \mathrm{u⃗}\right)}{\partial t}+\nabla \cdot \left(\rho \mathrm{u⃗}\mathrm{u⃗}\right)=-\nabla p+\nabla \cdot \mu \left[\nabla \mathrm{u⃗}+{\left(\nabla \mathrm{u⃗}\right)}^{T}\right]-{\mathrm{F⃗}}_{\mathrm{sf}}+\rho \mathrm{g⃗}$$

In this equation, *u⃗* is the velocity field, ∇*p* is the variation of pressure, and *F⃗*_*sf*_ represents the **surface tension force**, which plays a crucial role in defining the capillary effects at the fluid’s interface, such as the shape of droplets and bubbles. The surface tension force is given by

12 $$F_{\mathrm{sf}}=\sigma \varkappa \delta \left(\varphi \right)\mathrm{n⃗}$$

σ is the surface tension coefficient

ϰ is the interface curvature

*n⃗* is the interface normal vector

and

13 $$\delta \left(\varphi \right)=\{\begin{array}{ll} 0 & |\varphi |\geq \alpha \\ \frac{1+\cos\left(\pi \varphi /\alpha \right)}{2\alpha } & |\varphi |<\alpha \end{array}$$

This formulation accurately captures the influence of surface tension on the momentum equation, impacting the overall flow dynamics near the interface.

The effect of **wall adhesion** is included in the model by modifying the normal surface in the surface tension equation to account for contact angles at the walls. The modified normal vector is expressed as

14 $$\mathrm{n̂}={\mathrm{n̂}}_{w}\;\cos\theta_{w}+{\mathrm{t̂}}_{w}\;\sin\theta_{w}$$

θ_*w*_ is the contact angle

*n̂*_*w*_ is the unit vector normal to the wall

*t̂*_*w*_ is the unit vector tangential to the wall

This modification allows the model to account for the interaction between the fluids and solid boundaries, which is essential for accurately predicting phenomena, such as droplet spreading, film formation, and contact line dynamics.

#### 3.2.4 Electric Potential Equation

The electric potential equation describes the distribution of electric potential within the fluids and is governed by the following partial differential equation:

15 ∇(σ∇φ)=0

φ is the potential

σ is the electrical conductivity

#### 3.2.5 Turbulence Equations

The standard *k*-ε turbulence model is widely used in computational fluid dynamics to predict the effects of turbulence by solving two key variables: the turbulent kinetic energy *k* and the dissipation rate ε. These two equations help determine how turbulence influences flow fields.

The transport equations for *k* (turbulent kinetic energy) and ε (dissipation rate) are given as follows:

**Turbulent kinetic energy (** ***k*** **) equation:**

16 $$\frac{\partial }{\partial t}\left(\rho k\right)+\frac{\partial }{\partial x_{i}}\left(\rho ku_{i}\right)=\frac{\partial }{\partial x_{j}}\left[\left(\mu +\frac{\mu_{t}}{\sigma_{k}}\right)\frac{\partial k}{\partial x_{j}}\right]+G_{k}+G_{b}-\rho \varepsilon -Y_{M}$$

**Dissipation rate (ε) equation:**

17 $$\frac{\partial }{\partial t}\left(\rho \varepsilon \right)+\frac{\partial }{\partial x_{i}}\left(\rho \varepsilon u_{i}\right)=\frac{\partial }{\partial x_{j}}\left[\left(\mu +\frac{\mu_{t}}{\sigma_{\varepsilon }}\right)\frac{\partial \varepsilon }{\partial x_{j}}\right]+C_{1\varepsilon }\frac{\varepsilon }{k}\left(G_{k}+C_{3\varepsilon }G_{b}\right)-C_{2\varepsilon }\rho \frac{\varepsilon^{2}}{k}$$

*G*_*k*_ *and G*_*b*_ are respectively the generation of turbulent kinetic energy due to the velocity gradient and the generation of turbulent kinetic energy due to buoyancy and *Y*_*m*_ is the effect of fluctuation dilatation on the dissipation rate. The base values of *C*_1ε_, *C*_2ε_, *C*_μ_, σ_*k*_, and σ_ε_ are kept as

$$\begin{array}{lllll} C_{1\varepsilon }=1.44, & C_{2\varepsilon }=1.92, & C_{\mu }=0.09, & \sigma_{k}=1.0 & \sigma_{\varepsilon }=1.3 \end{array}$$

### 3.3 Fluid Properties

For the 3D model, most of the properties used were based on the literature^4,6,8,11–14^ and are summarized in Table 3.

**Table 3:** Properties of Fluids at 965 °C

**fluid**	**density** **[kg/m** ^**3**^ **]**	**viscosity** **[kg/(m·s)]**	**electrical** **conductivity [S/m]**
**CO**_**2**_	0.4	1.37 × 10	10^–5^
**electrolyte**	2050	0.00256	220
**liquid aluminum**	2370	0.00127	3.5 × 10^6^

In a similar way, the following surface tensions were used.

The values presented in Tables 3 and 4 are nearly the same as those used in our 2D model, which was presented at the 41st International ICSOBA Conference.^10^

**Table 4:** Surface Tensions of Fluid Combinations

**fluid #1**	**fluid #2**	**surface tension** [N/m]
**CO**_**2**_	**electrolyte**	0.132
**liquid aluminum**	**CO**_**2**_	0.56
**liquid aluminum**	**electrolyte**	0.8

Finally, a contact angle of 110° is applied.^8^ This angle is very important, as it has a major impact on the force with which bubbles are held at the anode surface.

### 3.4 Geometry

The model represents a three-dimensional anode in an electrolytic cell with two slots on the long side of the anode. Figures 2 and 3 show the 3D model.

**Figure 2:**
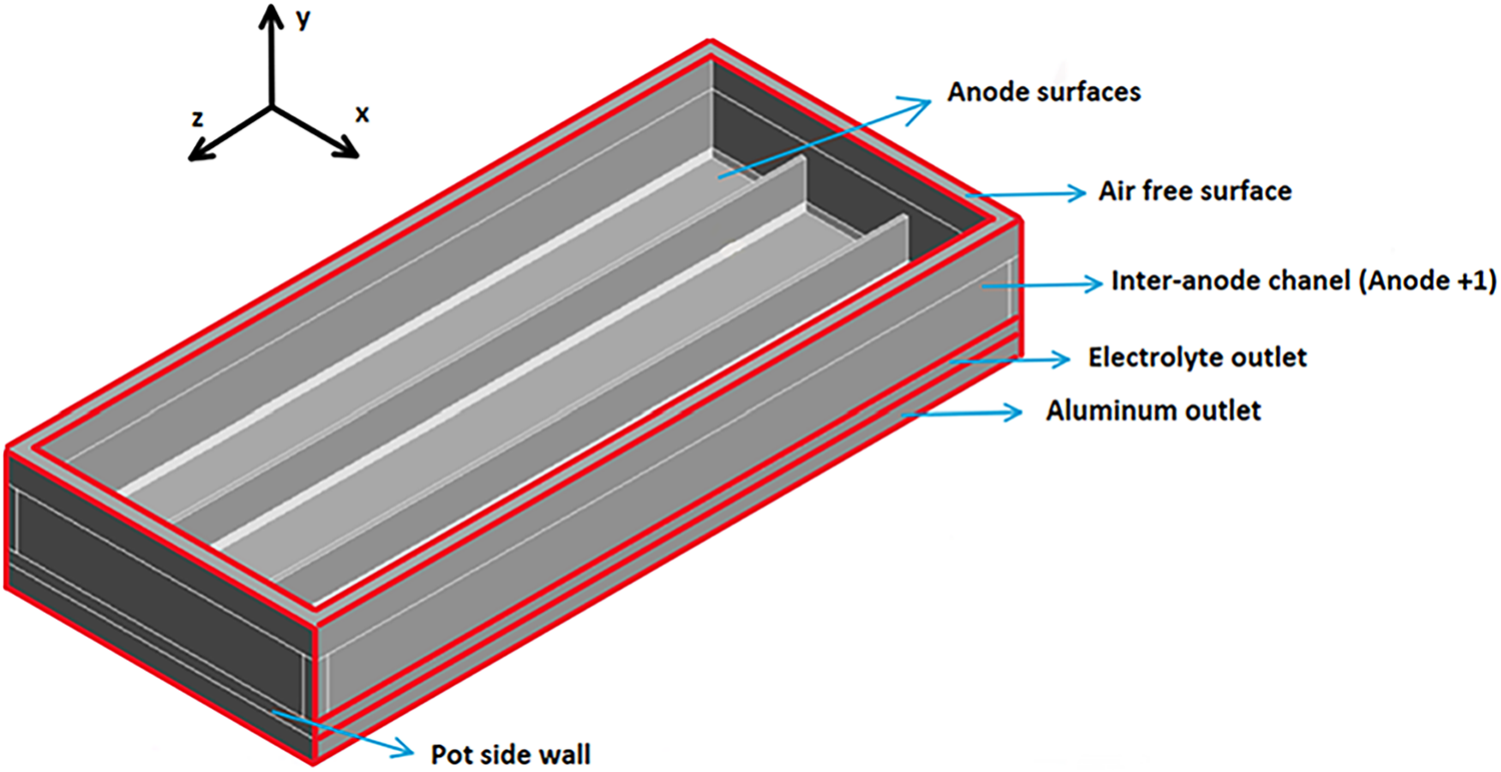
Isometric view of the model.

**Figure 3:**
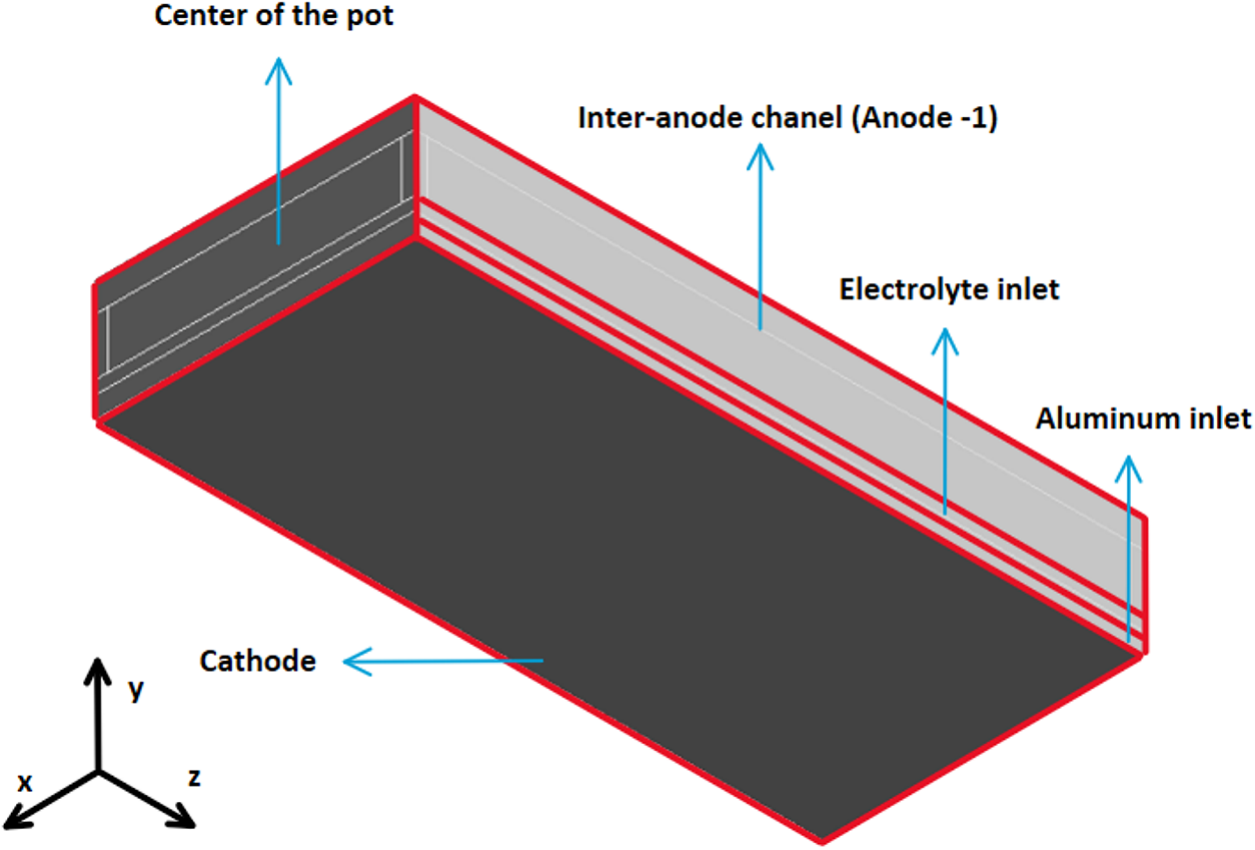
Back view of the model.

As shown in Figures 2 and 3, the cell’s central and lateral channels run parallel to the short side of the anode, while the interanode channels run parallel to the long side of the anode. The two channels crossing the anode represent the slots. Finally, the electrolytic bath is found under the anode surface, and then liquid metal is located on the cathode. The dimensions used are typical values from industry found in the literature.

- Slots width: 10 mm
- Slots height: 100 mm
- Center channel width: 30 mm
- Side channel width: 30 mm
- Interanode width: 30 mm
- ACD: 30 mm
- Liquid aluminum depth: 50 mm
- Bath height: 160 mm
- Anode length: 1600 mm
- Anode width: 670 mm
- Anode inclination in *x* direction: 1.5°
- Anode inclination in *z* direction: −1.5°

In industrial environments, the height of liquid aluminum is greater than 50 mm. However, this reduced height was chosen to reduce the simulation time.

### 3.5 Boundary Conditions

The boundary conditions were chosen to reproduce the flow in the cell as accurately as possible. Thus, for each boundary, fluidic and electrical conditions are determined.

Table 5 shows the conditions attached to each of the boundaries. The visual representation of the boundaries is presented in Figures 2 and 3.

**Table 5:** Boundary Conditions Used

**boundary**	**fluidic condition**	**electrical condition**
anode surfaces	no slip wall	0 V
cathode	free slip wall	imposed current density
side wall	no slip wall	insulated
center of the pot	symmetry	symmetry
interanode channels	symmetry	symmetry
aluminum inlet	mass flux inlet	insulated
aluminum outlet	mass flux outlet	insulated
electrolyte inlet	mass flux inlet	Insulated
electrolyte outlet	mass flux outlet	Insulated
air-free surface	pressure outlet	Insulated

As shown in Table 5, the electric potential is imposed at the anode surfaces and the current density at the cathode. This choice is made in order to have a realistic current distribution under the anode. Indeed, since the voltage is imposed at this point and not the current density, the latter can be distributed in such a way as to minimize the resistance imposed by the presence of the bubbles.

One can also note the presence of aluminum and electrolyte inlet and outlet conditions. These conditions are used to simulate the flow of aluminum generated by MHD. Thus, by imposing this motion, the effect of MHD flow on the electrolyte and bubbles is represented. Finally, the pressure outlet allows the generated CO_2_ to evacuate freely.

### 3.6 Modeling of the Anode Bottom Surface

A strategy inspired by Kiss and Poncsák^15–17^ was used for the bubble generation model. Using this method, a standard area for the elementary cells was determined, and then the bottom surface of the anode was divided into approximately 13,000 elementary cells of the same dimensions. The mesh element at the center of the elementary cell acts as the nucleation site, where CO_2_ is generated. Figure 4 illustrates the division of the bottom of the anode into elementary cells.

**Figure 4:**
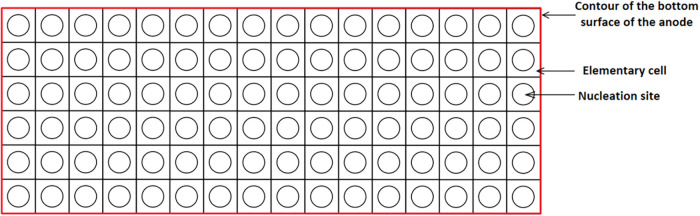
Division of the anode’s bottom surface.

To manage the production of bubbles in accordance with this method, a user-defined function (UDF) was made. This function uses Faraday’s law to calculate the mass of CO_2_ generated in each nucleation site as a function of the localized electrical current “*i*” in the elementary cell.

18 $$m_{{\mathrm{CO}}_{2}}=\frac{i\cdot t\cdot M}{z\cdot F}$$

where:

*F* is the Faraday’s constant (96 500), C/mol

*i* is the electric current, A

*t* is time, s

*z* is the number of electrons involved in reaction 4 ^16^

*M* is the molar mass of CO_2_, kg/mol

*t*, *M*, *z*, and *F* are all constants in this context. The mass generated therefore depends only on the amount of current flowing locally into the elementary cell.

## 4 Results and Discussion

In this work, six cases were simulated. Case #1 corresponds to the reference case for studying the influence of ACD, the direction of MHD flow, and the width of the channels on different indicators. It has an ACD of 30 mm, an MHD flow parallel to the anode’s width (in the positive x direction), and channel width of 30 mm.

Table 6 summarizes the parameters of all of the cases.

**Table 6:** Cases Studied

**case #**	**description**	**ACD (mm)**	**MHD-induced flow**	**channel width (mm)**
1.	base case	30	parallel to center channel	30
2.	30 mm ACD	**35**	parallel to center channel	30
3.	40 mm ACD	**40**	parallel to center channel	30
4.	MHD flows from side to center channel	30	**from side to center channel**	30
5.	MHD flows from center to side channel	30	**from center to side channel**	30
6.	increased channel widths	30	parallel to center channel	**50**

In each of these cases, even when the MHD direction is varied, the velocity used remains the same, i.e., 10 cm/s, and has been chosen from simulations outside the scope of this research.^18^

A slight inclination of the anode (1.5°) is also added in the *x* and *z* directions, where the higher side is on the positive side for the *x* direction and on the negative side for the *z* direction. The inclination used is based on the results of another study that investigated the effect of MHD on the metal’s deformation.^18^ The hypothesis is that the anode is deformed to the same angle as the metal pad beneath it. Thus, for every case, the anode inclination corresponds to a permanent deformation of the BMI.

The following analysis is presented in three subsections:

1. Cases #2 and #3 have the purpose of finding out the effect of ACD on the output parameters. Steps of 5 mm are made, and the range from 30 to 40 mm is studied.
2. The aim of Cases #4 and #5 is to determine the effect of MHD direction on the bubble flow. The results can tell a lot about the behavior of fluids as a function of the position of the anode in the cell, since the direction of MHD varies within the pot.
3. For Case #6, the width of the center and the side channel as well as the distance between the anodes was increased from 30 to 50 mm to see the effect on cell’s response.

### 4.1 General Observations

The videos produced in these simulations show some interesting phenomena. In this way, Figures 5 and 6 show the CO_2_ bubbles for the “Base case” after 6 s of simulation.

**Figure 5:**
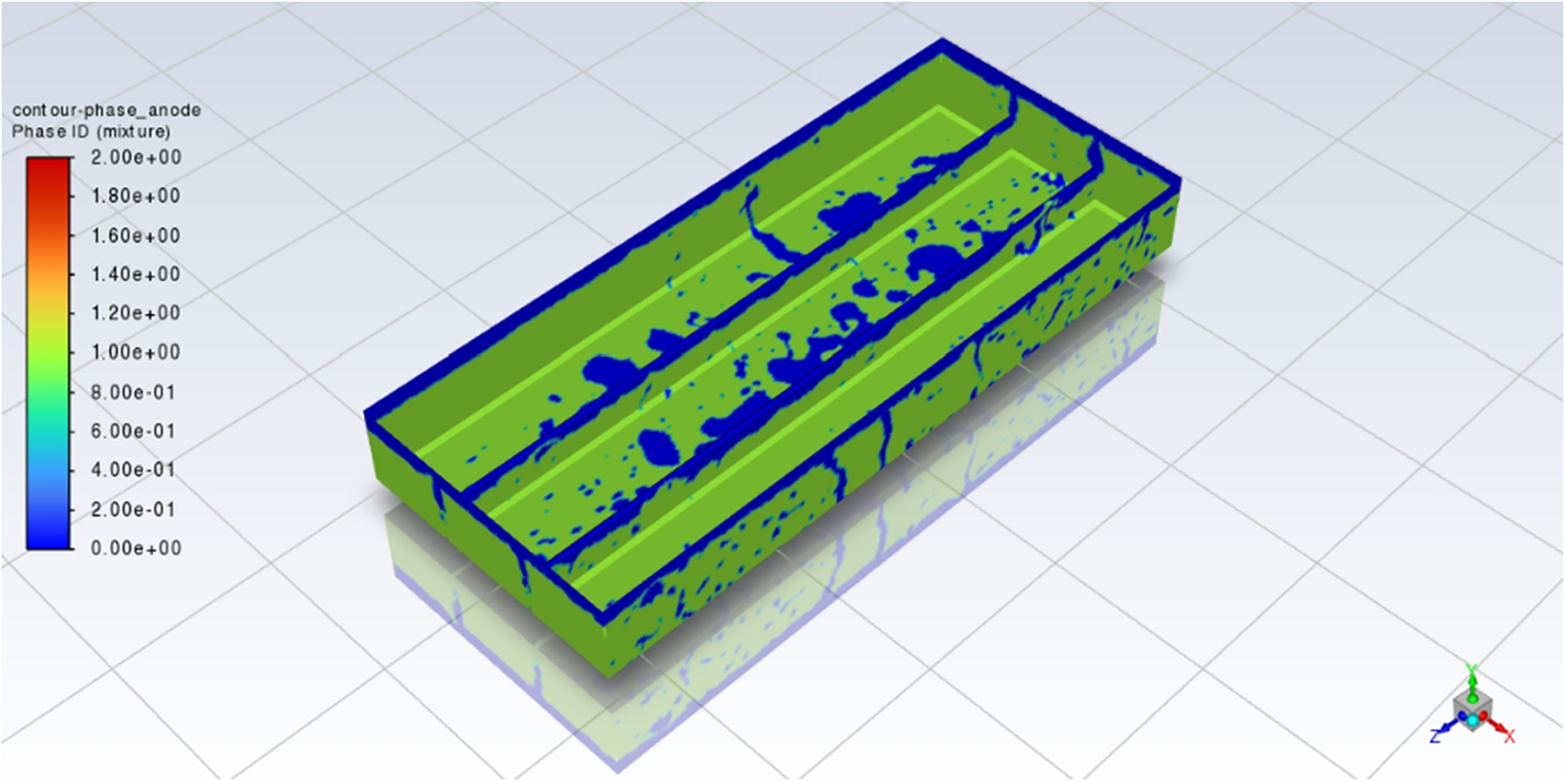
Phase of the anode contour of case #1 at *t* = 6 s.

**Figure 6:**
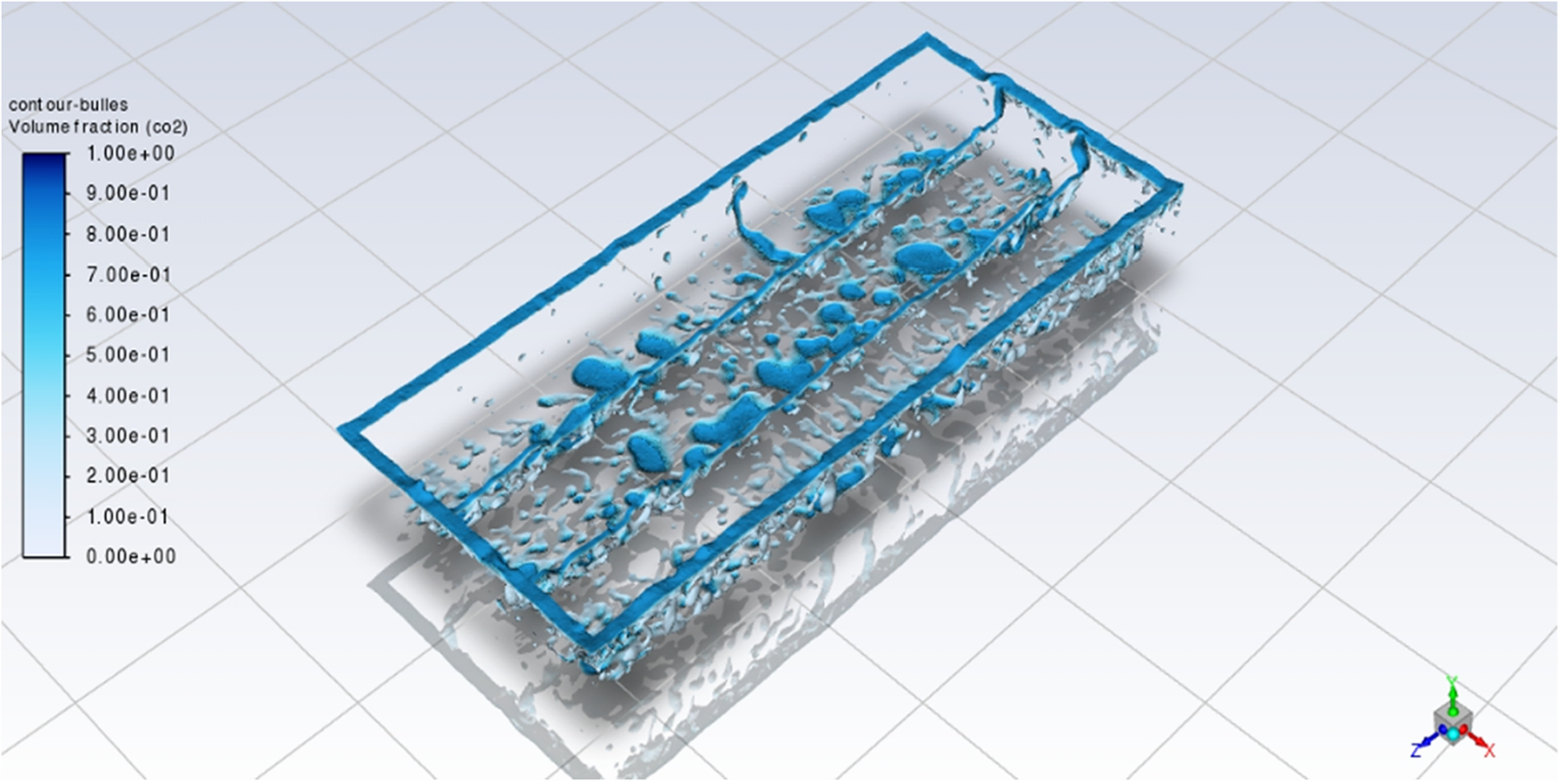
Iso-surfaces of CO_2_ in case #1 at *t* = 6 s.

In these two figures, CO_2_ bubbles of various sizes are trapped under the anodes. In both figures, bubbles are represented by blue areas. They show bubbles evacuated through the various channels. In particular, the largest bubbles are seen near the slots and the interanode in positive x direction. These three zones correspond to the preferential escape zones for the bubbles. In fact, these channels are favorable for bubble escape, since gravity and the direction of MHD act on bubbles to move toward this direction. This effect corresponds to the observations made by Zhan et al.,^5^ as described in the literature review.

On a different note, a cross-sectional view of the *z*-axis direction in the slot is represented in Figure 7.

**Figure 7:**
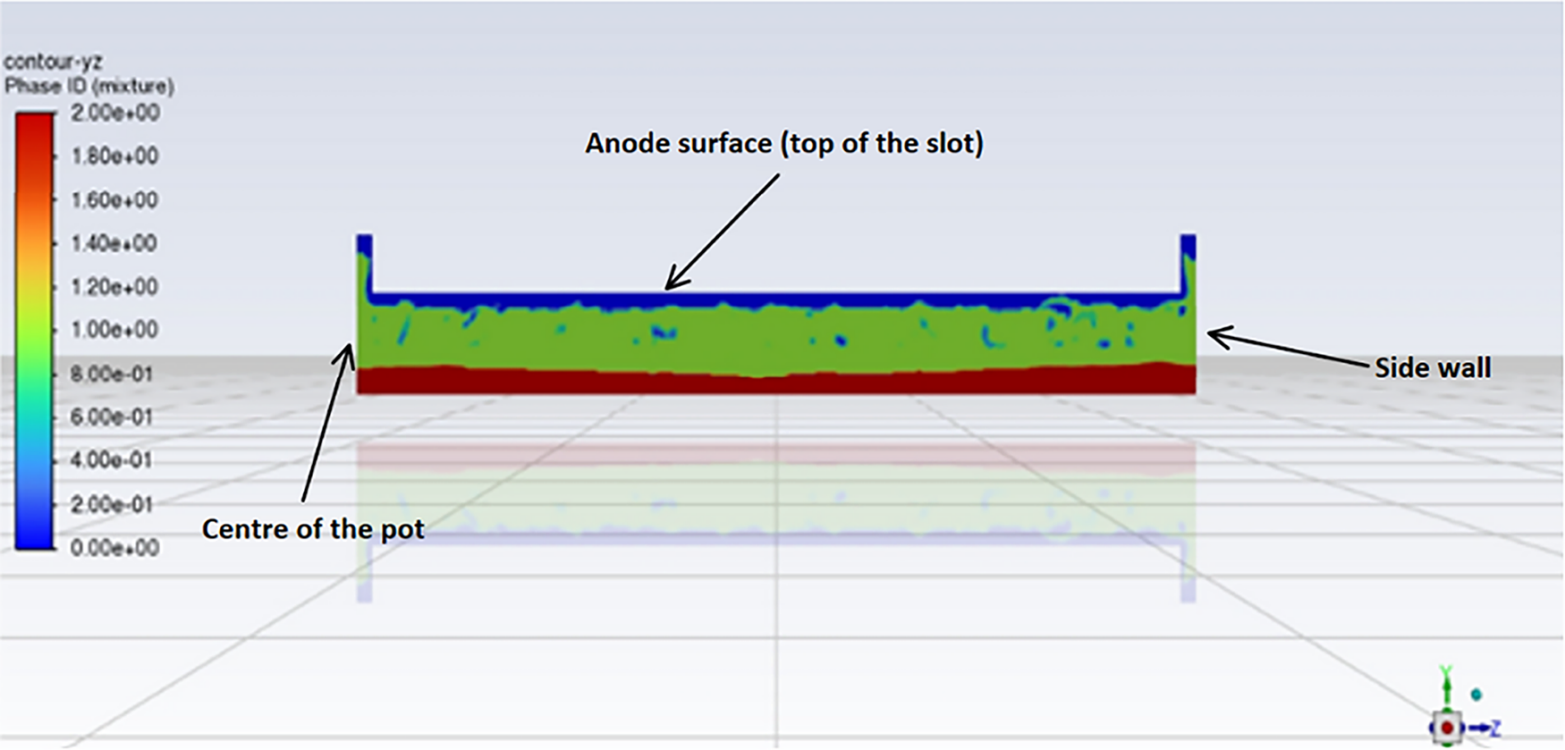
Section view of slot 1 after 6 s of simulation for case #1.

In this figure, red represents liquid aluminum, green represents the electrolytic bath, and blue is the CO_2_ gas. The figure shows a bubble layer that is trapped in the slot before being released. The bubbles then escape on either side of it. The gas layer builds up at the start of the simulation before rapidly reaching an equilibrium point.

### 4.2 Effect of ACD

In industry, the desire would be to control and even reduce ACD as much as possible in order to enhance the control efficiency and ultimately reduce energy consumption. To do this, it is important to understand the impact of a reduction in ACD in order to make the optimal choices.

#### 4.2.1 The Preferential Path of Bubbles

The bubble evacuation path has a major effect on cell performance. In particular, if the bubbles escape easily, then the tension created by the bubble resistance is reduced. Similarly, bubbles escaping toward the center will promote alumina dissolution, while those escaping toward the side will increase heat transfer to the outside of the cell.

Thus, for this analysis, bubble escape is divided into two parts.

Part 1: Bubbles escape from the bottom surface of the anode in one of these six directions:

1. Toward anode – 1 (− × direction)
2. Toward slot 1
3. Toward slot 2
4. Toward anode +1 (+ × direction)
5. Toward the side channel
6. Toward the center channels
  1 to 4 represent the escape path in the *x* direction while 5 and 6 are escape path in the *z* direction.
Part 2: The bubbles escape the slots
7. Toward the side channel
8. Toward the center channel

Table 7 shows the effect of the ACD on the fraction of bubbles escaping through each zone.

**Table 7:**
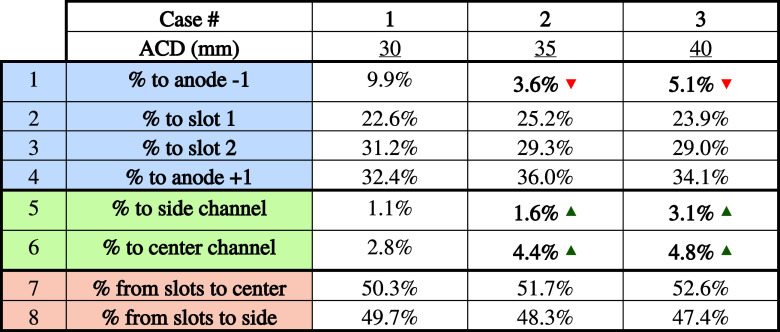
Bubble Path as a Function of ACD

In the table, the red and green arrows show variations greater than 20% compared with the base case.

First, in Table 7, rows 1 to 6 show all possible evacuation paths for a bubble below the anode. Then, rows 7 and 8 present the two possible directions for the evacuation of bubbles that are in the slots.

Looking first at the “Base case” (30 mm), the bubbles are much more likely to escape through the long channels, i.e., the slots and interanode channels. In fact, 96.1% of bubbles escape through these 4 channels (1 to 4), while only 3.9% escape through the anode ends (5 and 6).

There are two reasons for this behavior:

1. The two main forces push the bubbles in this direction. The flow due to MHD in this case is along with the positive *x*-axis. In addition, a gravity of 1.5° acts in the same direction.
2. The width of the blocks is much shorter than their length. In particular, since the anode is divided into three by its two slots, it results with three surfaces that are 1600 mm long and 216 mm wide. The bubbles therefore have a much shorter path to evacuate along the width of the anode rather than the length.

In terms of evacuation through channels 5 and 6, there is a higher proportion to the center channel than to the side channel. This is due to the 1.5° angle also present in the negative z direction, which pushes the bubbles toward the center rather than the side wall.

Columns 7 and 8 of the “Base case” in Table 7 show that the same proportion of bubbles is sent from the slots to the center or to the wall. This suggests that the angle under the anode (the slot has no angle) has little influence on the bubble’s path of escape when it is returned to the slot.

No clear behavior is observed in Table 7 for rows 1 to 4. Although for row 1, the differences are greater than 20%, the difference remains small in absolute terms. Moreover, the behavior is not reflected in rows 2 to 4, which all have similar values from one case to the other. This is to be expected since similar forces act on the underside of the anode in all three cases.

Finally, although the differences between case #1 and cases #2 and #3 for channels 5 and 6 are relatively large, the absolute difference remains small. The increase may therefore be due to the randomness of the cell’s behavior.

#### 4.2.2 The Tension

Voltage is directly proportional to energy, which explains the importance of reducing it as much as possible. Indeed, electrical energy consumption represents one of the major operational expenses of an aluminum smelter.

For each of the simulated cases, the tension was computed at each instant, giving distributions, as shown in Figure 8.

**Figure 8:**
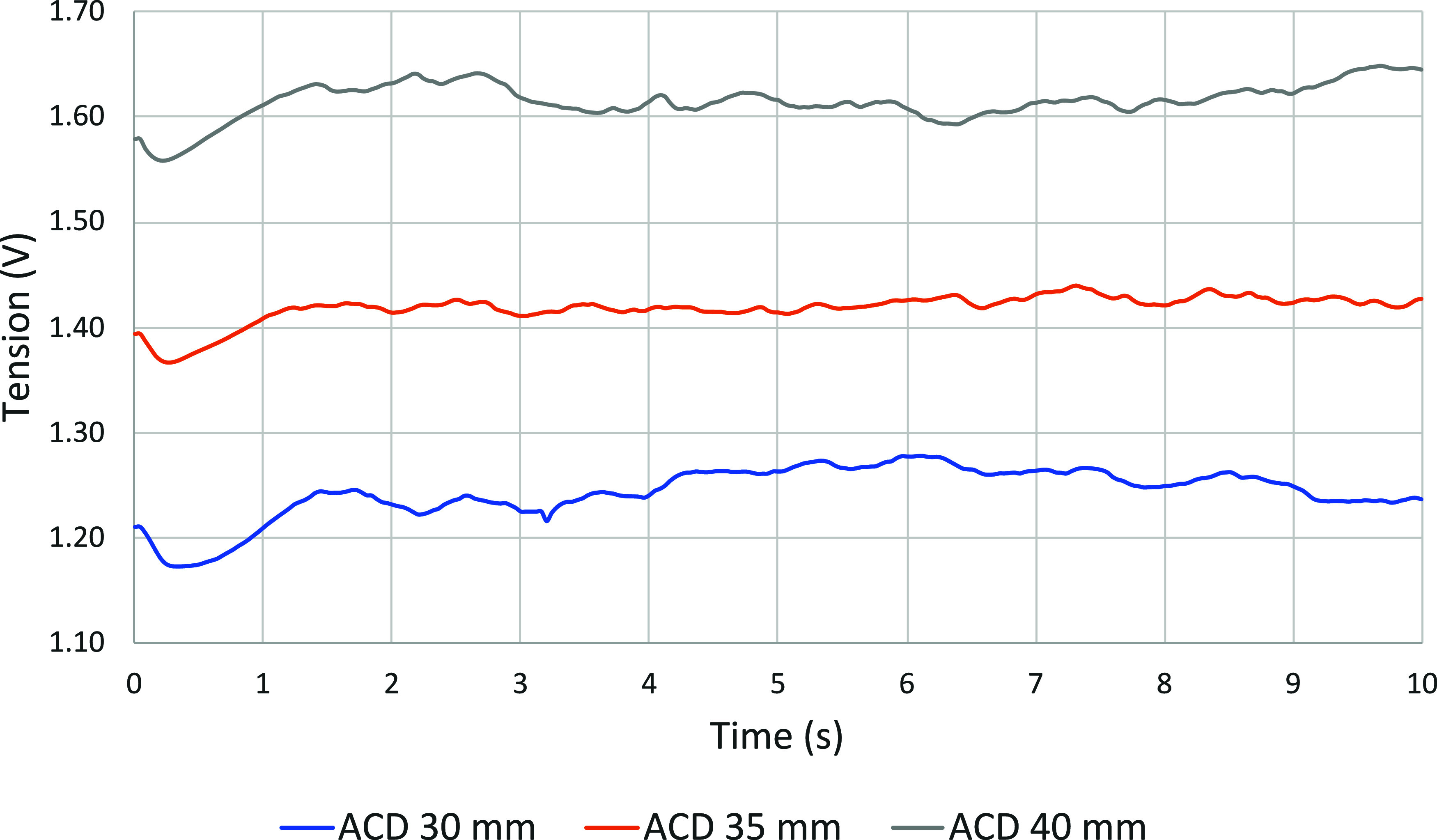
Tension through time for cases nos. 1, 2, and 3.

Figure 8 shows an initial stabilization period between 0 and 2 s during which the values behave differently than when the system is in equilibrium. On the other hand, as shown in Figure 11, the deformation of the BMI takes around 4 s to become stable. With this in mind, the decision was taken to consider seconds 4 to 10 for the analysis.

Voltage variations are mainly due to the accumulation and evacuation of CO_2_ bubbles, which means that these data can provide a better understanding of the accumulation of bubbles under the anode, its escape frequency, and the voltage amplitude involved.

Figure 9 presents the tension drop between the anode and the cathode for the three cases with varied ACD. The value presented for each case corresponds to the average voltage between the 4th and 10th seconds.

**Figure 9:**
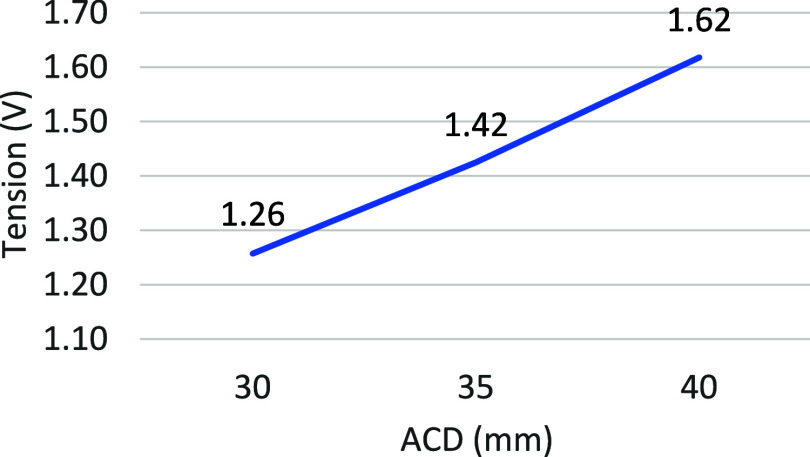
Effect of ACD on the mean tension.

The increase in voltage is proportional to the increase in ACD because the voltage is proportional to resistance, which in turn increases with the thickness of the medium to be crossed. The slightly more pronounced voltage rise for the second ACD increase (35–40 mm) is potentially due to the reduction in the lateral surface area of the anode in the liquid bath. In fact, from one simulation to the next, the total bath height remains the same, meaning that immersion is slightly reduced when the ACD increases.

Part of this difference in variation may also be due to the randomness of the system and the limited simulated time period of just 10 s.

Finally, it could also show that bubbles escape with a little more difficulty when the underside of the anode is farther from the pad metal.

#### 4.2.3 The Bath–Metal Interface Deformation

Deformation of the bath–metal interface is a key output parameter, since it is mostly what limits the reduction of the ACD. If the deformation of the BMI is too important, the liquid aluminum gets closer to the gaseous CO_2_ present under the anode, triggering the reoxidation reaction, which is undesirable in the cell. Consequently, a more uneven BMI interface will force liquid aluminum closer to the bubble interface and is such an indicator of potential decrease in current efficiency.

Figure 10 shows the deformation of the BMI at 6 s for the “Base case”.

**Figure 10:**
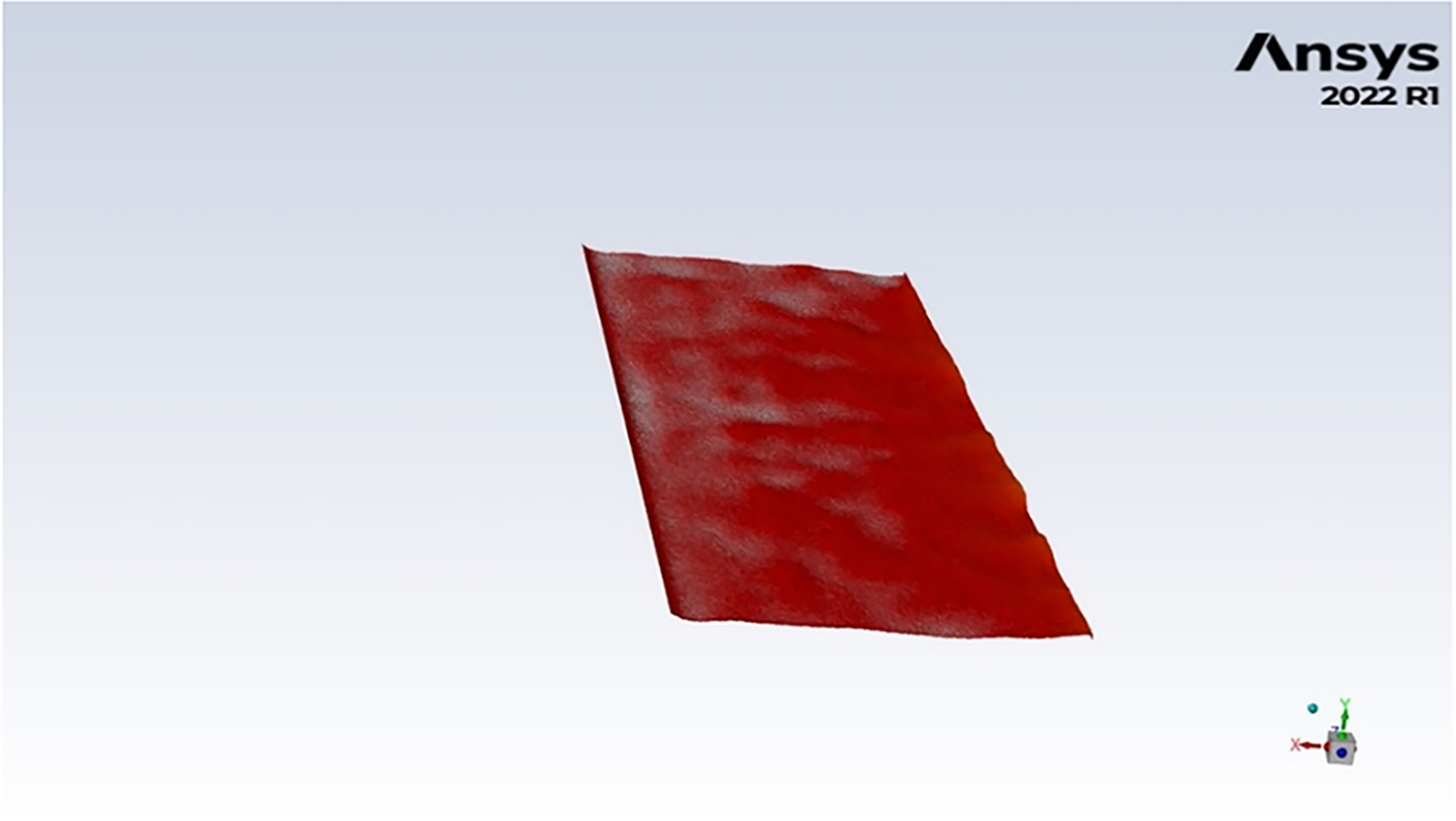
BMI deformation at 6 s for case #1.

To quantify the average deformation of the bath–metal interface, a regular grid is defined in the xz plane with a spacing of 1 cm in both directions, resulting in a dense array of sampling points. At each grid node, the local height of the interface is measured and compared to the resting height of the undisturbed bath–metal interface. The absolute difference between the measured and reference heights is calculated for each point, and the average of these values is evaluated at every time step. Equation 19 shows how this calculation is done.

19 $$\mathrm{deformation}=\frac{1}{n}\sum_{i=1}^{n}\left|h_{i}-h_{0}\right|$$

where *n* is the number of grid nodes, *h_i_* is the interface height at this location, and *h*_0_ is the reference height.

This calculation results in Figure 11 for cases #1 to #3.

**Figure 11:**
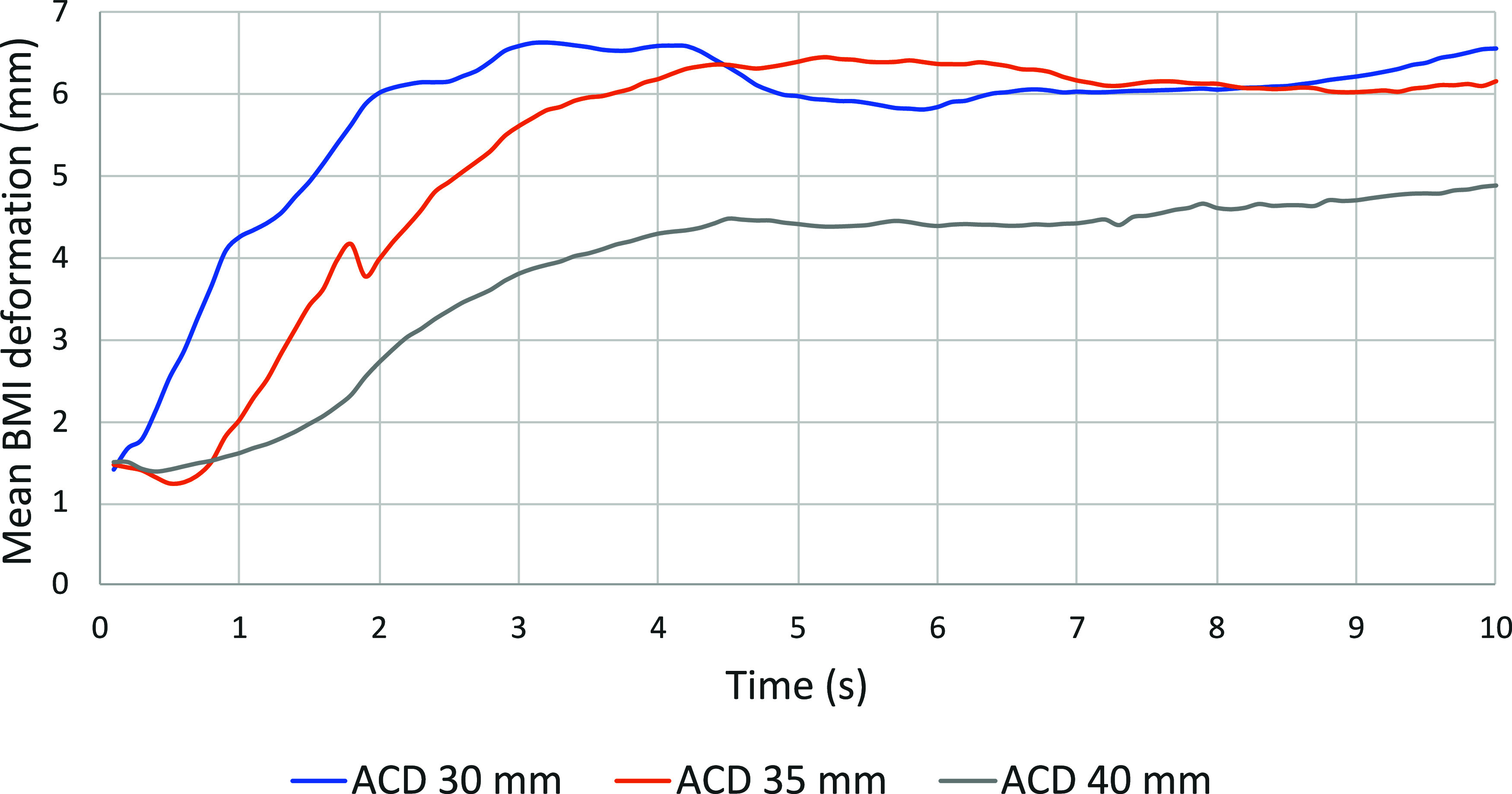
Mean BMI deformation for cases 1 to #3.

In this process, the bath–metal interface is deformed following the escape of gas bubbles from underneath the anode as it generates a localized displacement of the fluids surrounding the bubble rising to the upper surface, which is rapidly filled by electrolyte. This upward movement of the bath propagates to the liquid aluminum located lower in the domain. It is this force acting on the liquids that disrupts and deforms the bath–metal interface. Figure 11 shows that with a larger ACD (40 mm), this deformation of the interface is less pronounced when the gas bubbles that create the initial disturbance are farther away from the interface, which allows for a longer distance to dissipate the effect of that drag force and mitigate its impact on the aluminum surface.

Following a mathematical measurement of the deformation of the metal pad at each time step, for every simulation, the average deformation between the 4th and 10th seconds is calculated in order to compare the cases with each other. Accordingly, the average deformation and standard deviation for these simulations have shown a BMI of 6.15 ± 0.24 for the 30 mm ACD, 6.21 ± 0.14 mm for the 35 mm ACD, and 4.53 ± 0.16 mm for an ACD of 40 mm.

The difference between case no. 1 (30 mm ACD) and case no. 2 (35 mm ACD) is negligible. Figure 11 shows that deformation appears to be lower for case #2 in the first 4 s, but when it reaches equilibrium, deformation for both cases is similar. However, there is a significant difference between the 35 mm ACD case and the 40 mm case. The average deformation is 1.68 mm lower, a reduction of 27%, which seems to demonstrate that there is a breaking point between 35 and 40 mm. This reduced deformation might allow the process to be more stable, with less reoxidation. To determine the exact behavior between 35 and 40 mm, a multitude of simulations would have to be performed with very short ACD intervals.

#### 4.2.4 The Turbulent Kinetic Energy

In a pot, it is necessary to direct the bubbles optimally to increase alumina dissolution in the central channel while limiting stirring in the side channel to avoid excessive heat exchange with the cell wall. Indeed, excessive thermal exchange with the wall may expose the cell lining materials and cause premature degradation of the cell lining. The turbulence kinetic energy is a good indicator of the difference between different operational cases.

Figure 12 shows the effect of ACD on the mean turbulence kinetic energy in the center channel and in the side channel.

**Figure 12:**
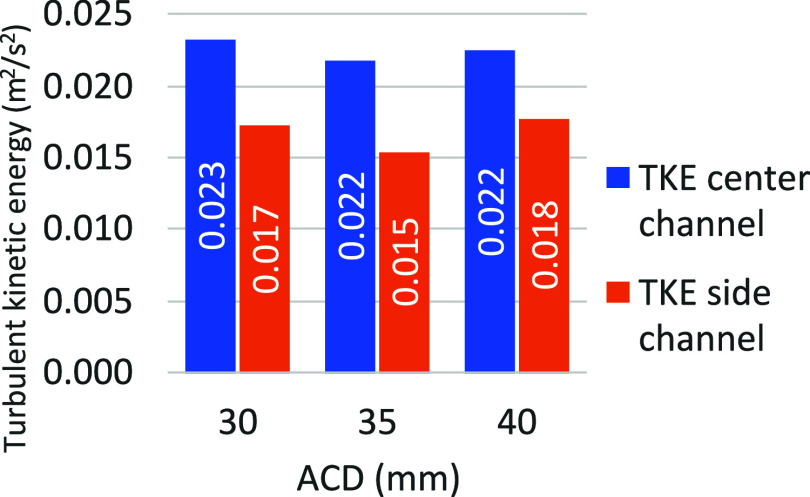
Effect of ACD on the mean TKE.

The results show very little variation, except for a very slight downward trend when the ACD is increased to 35 mm, but it disappears with the second increase in the ACD to 40 mm.

A key observation from the figure is the significant effect of the angle of the underside of the anode on TKE. In fact, the kinetic energy of turbulence is around 30% higher in the center channel than in the side channel, due to the angle, which causes more gas to escape toward the center. In fact, the angle given to the anode in this simulation assumes that the anode deforms into the same shape as the metal pad beneath it. This means that the angle of the anode can promote better dissolution of the alumina in the central channel and reduce heat exchange with the side wall.

### 4.3 Effect of the MHD Direction

The movement of liquid aluminum at the bottom of the cell is not uniform. The MHD forces impacting the movement of the metal vary according to the position of the cell. In some locations, movement is in the x direction, and elsewhere, it can be in the *z* direction. In fact, the orientation varies by 360° in the horizontal plane since the flow is in the form of two complete asymmetrical loops.^18^ The aim is therefore to verify the impact of the direction of the pad movement on the gas flow.

#### 4.3.1 The Preferential Path of Bubbles

In this section, the direction of metal flow varies from one case to another. However, the anode angles remain constant between the cases. Figure 13 shows a view from the top of the cell to give a better understanding of the different MHD directions that have been studied. The orange outline rectangle represents the modeled anode block as reference. Knowing that the MHD flow takes the form of two loops over half the pot, simulations 1, 4, and 5 correspond to simulating three anodes placed at different locations in the cell.

**Figure 13:**
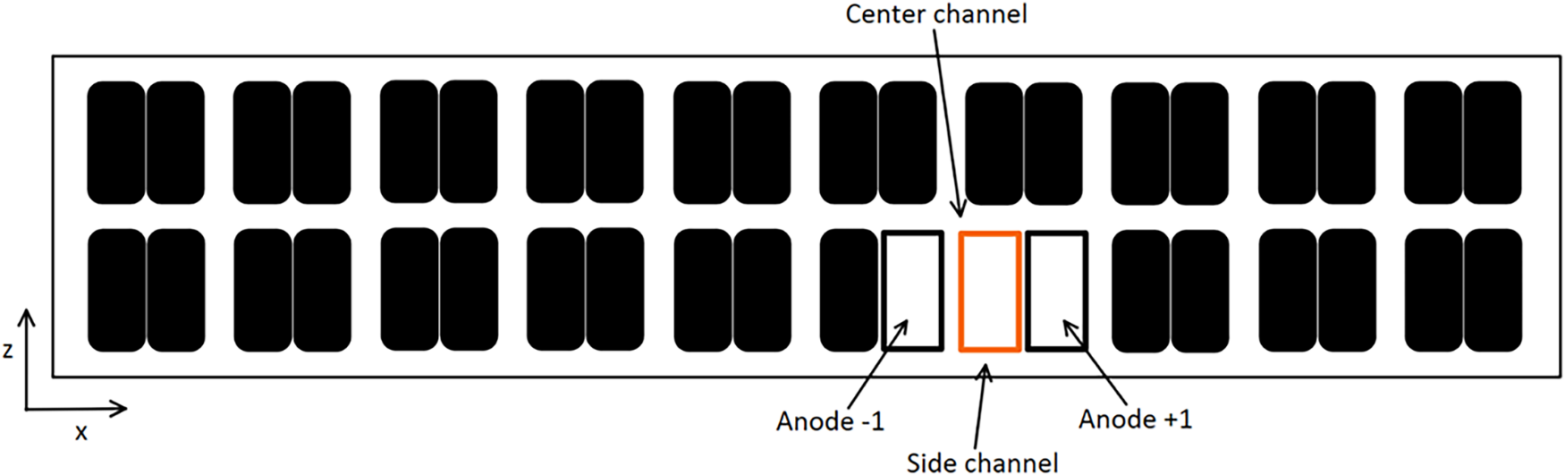
Top view schematic of the cell.

Table 8 shows the effect of the metal flow direction on the bubble evacuation path.

**Table 8:**
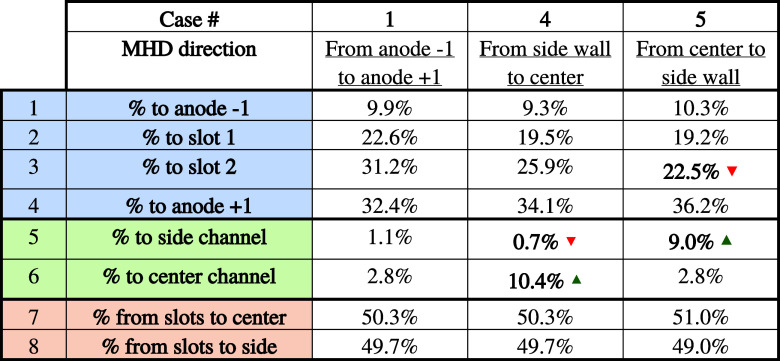
Bubble Path as a Function of MHD Direction

In the table, the red and green arrows show variations greater than 20% compared with the base case.

The results show that the direction of metal flow has an important effect on the bubble escape path. Indeed, when MHD acts from the side wall toward the center of the cell (case #4), there is a large increase in the fraction of bubbles evacuating toward the central channel, rising from 2.8 to 10.4%. It means that around 7.6% of the total volume of bubbles are no longer escaping through the slots or the interanode channels and now escape to the center channel of the cell. This change in the evacuation path will promote alumina dissolution as mixing throughout the central channel is enhanced by the presence of these bubbles.

Similarly, in case #5 with MHD acting from the center to the side wall, this time there is an increase in the proportion of bubbles escaping from the underside of the anode directly to the side wall (+7.9%). This again demonstrates the impact of the MHD on bubble flow.

Then, rows 7 and 8 show that the direction of MHD has no impact on the behavior of bubbles at the top of the slots, since in each case around 50% of the gas escapes on each side. This suggests that when the grooves are 10 cm deep, the gases escaping from them are too far from the flow of metal at the bottom of the pot to be significantly impacted by it.

These observations show how important the direction of metal flow is to bubble evacuation since its effect is even greater than the effect of gravity created by a 1.5° angle. A more thorough analysis taking into consideration the ACD, slot height, velocity of the metal flow, and its direction may prove to be even more insightful, especially to understand the heterogeneous behavior of bubble-induced flow resulting from a nonuniform MHD movement in the cells.

#### 4.3.2 The Tension

Figure 14 shows the evolution of the tension for cases # 1, 4, and 5, where the MHD-induced flow direction was varied. Figure 15 presents the mean value of the tension for the three cases.

**Figure 14:**
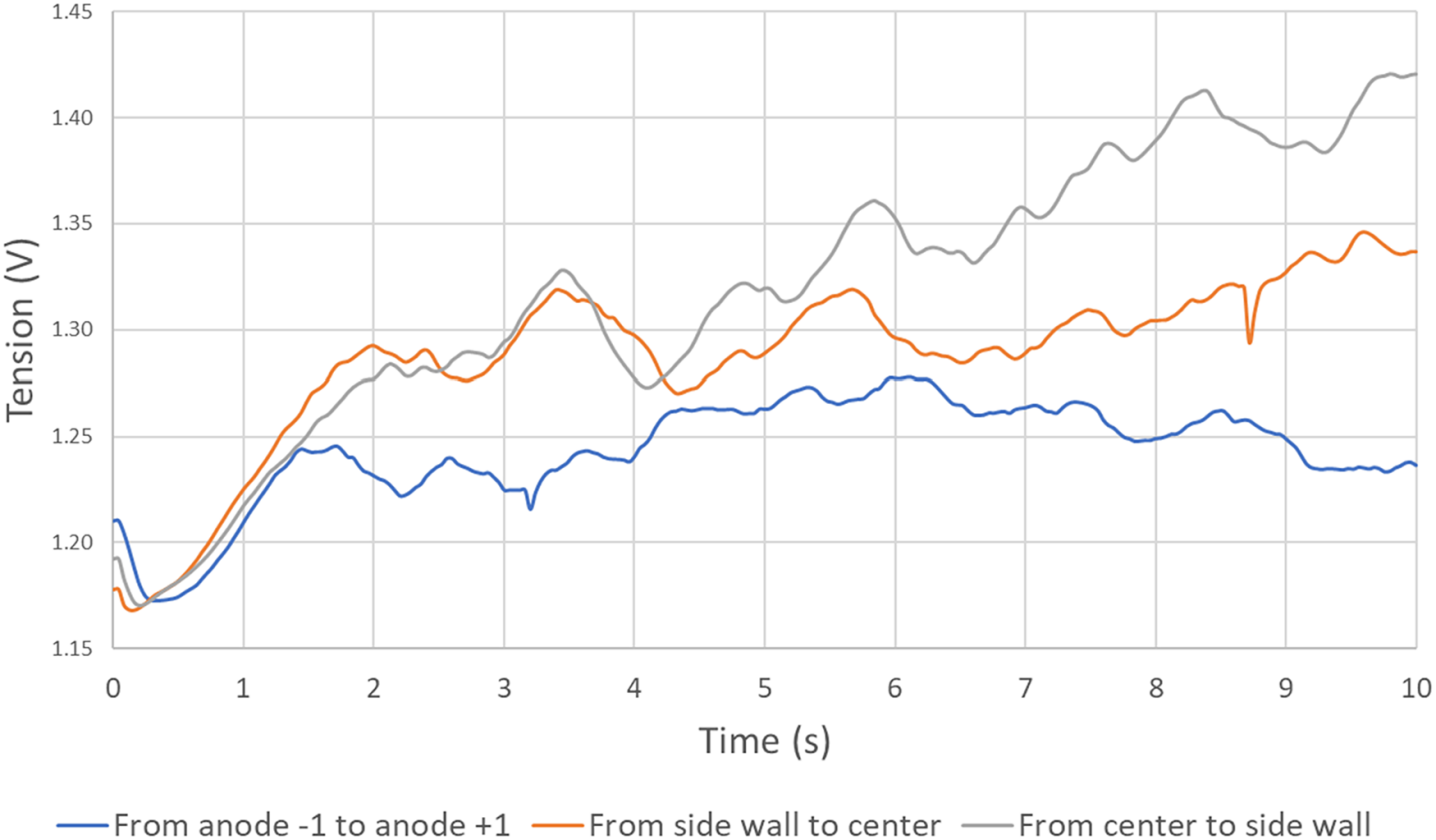
Evolution of the tension in time for cases 1, 4, and 5.

**Figure 15:**
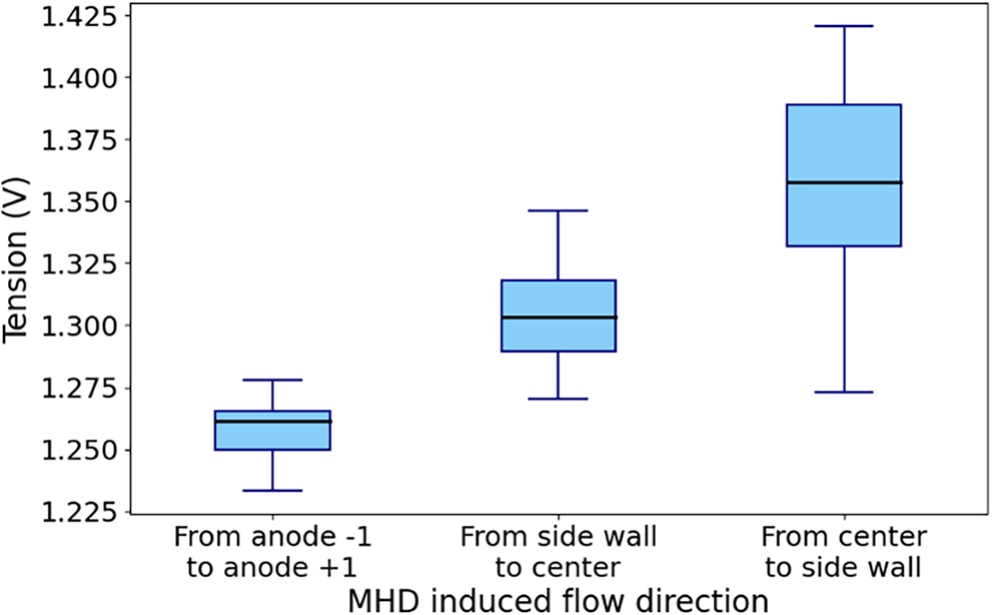
Boxplot of the effect of the MHD direction on the tension.

Figures 14 and 15 show a significantly lower voltage in the case where the MHD generates motion in the direction of the anode width rather than in the direction of the anode length. This difference (up to 90 mV on the mean voltage) is due to the fact that in case #1, fluid flow in the *x*-axis pushes the bubbles into the slots, whereas in the other two cases, MHD pushes the bubbles toward the central or lateral channel. However, thanks to the slots, when bubbles are pushed along the *x*-axis rather than the *z*-axis, they reach an evacuation channel more quickly, resulting in lower bubble coverage under the anode. While this value may appear small, given that an electrolysis cell operates at about 4.2 V, such an augmentation on the mean tension (90 mV) may represent a required power increase on the order of 2%.

Moreover, in the case where the MHD acts toward the side wall, the increase in voltage due to bubble build-up is much greater, as the MHD acts against the buoyant force induced by the anode angle.

The analysis shows that the direction of the MHD flow under the anode has a major impact on its gas coverage. At the same time, the analysis also indirectly shows the effectiveness of slots since dividing the anodic bottom surface width facilitates the escape of the gas.

#### 4.3.3 The Bath–Metal Interface Deformation

Figure 16 shows the average BMI deformation as a function of the MHD direction.

**Figure 16:**
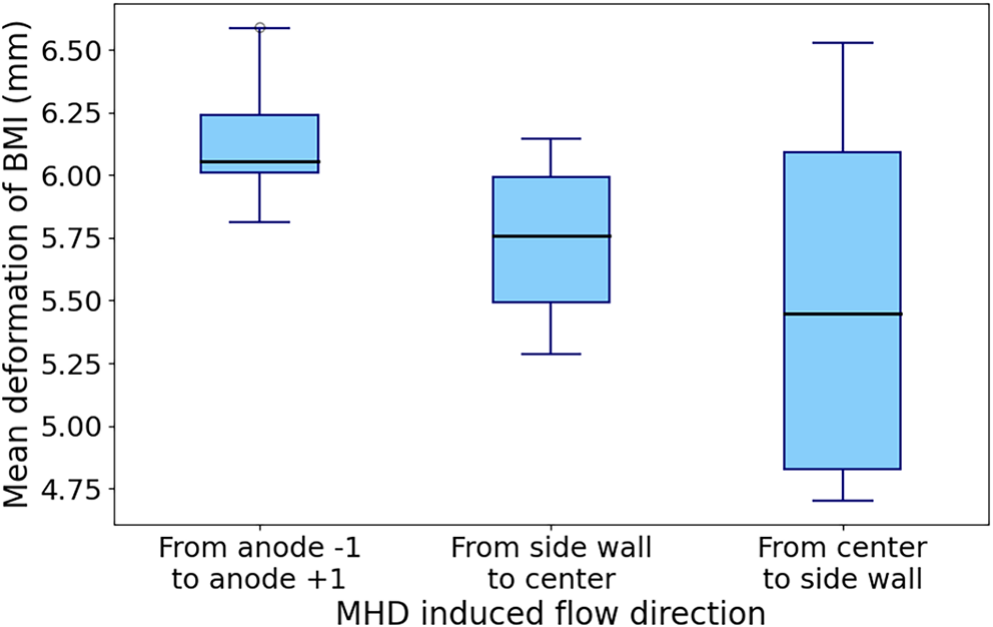
Effect of the MHD direction on the mean BMI deformation.

In Figure 16, the “base case” has the highest median and a more constant BMI deformation. A likely hypothesis is that the bubbles escaping through the slots create greater fluid entrainment, given that these channels are narrower than the center and side channels (10 vs 30 mm). Indeed, in the reference case, around 54% of the total volume of bubbles escape through the slots, whereas in cases #4 and #5, it is only 45.4 and 41.7% respectively.

While the difference in the median BMI deformation is at most only 0.6 mm, it could be a mistake to disregard that variation as negligible since many smelters reported changes in their nominal ACD by only a few mm with sustainable gain in specific energy consumption. When considering the heterogeneous aspect of MHD in the cell, these results highlight the dynamic nature of the process and pinpoint the areas of the cell where higher localized instability are expected from the turbulent nature of the fluid movement in the ACD.

#### 4.3.4 The Turbulent Kinetic Energy

The turbulent kinetic energy in the lateral and central channels are outputs that are likely to be impacted by the MHD flow direction since the bubble evacuation path is modified. Figure 17 shows the magnitude of this effect.

**Figure 17:**
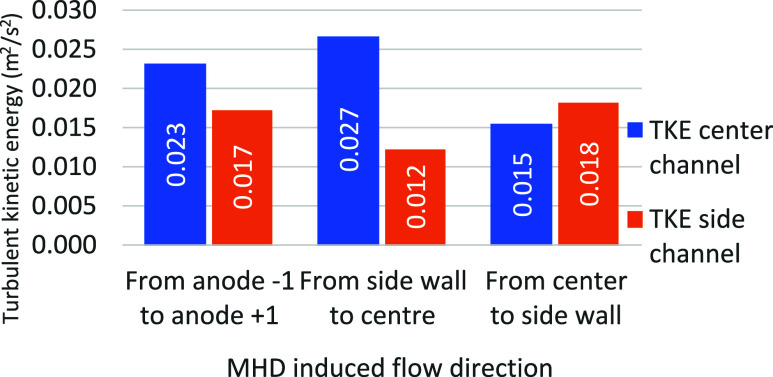
Effect of the MHD direction on the mean TKE.

As expected, in the case where MHD acts from the side channel to the center channel, the TKE in the side channel is decreased while that in the center channel is slightly increased. Similarly, the opposite is true when the MHD moves from the center of the cell to the side wall.

In the case of MHD going to the central channel, an increase in bubble escape directly to the central channel of 7.6% generates an increase in TKE of 17.3%.

Depending on the position of the anode in the cell, the direction of the MHD flow will be different. The results therefore show that the position of the anode in the cell can have a crucial impact on alumina dissolution and erosion of the side wall. Indeed, by correlating these results with the modelized MHD flow of the cell, it is possible to quantitatively define which feeders’ position have a significant dissolution advantage based on the bath mixing occurring in their vicinity. Similarly, it is possible to pinpoint the precise anode positions within the cell that are inherently disadvantaged due to the MHD-induced bubble flow, which reduces wall protection and increases the risk of metal tap out.

### 4.4 Effect of the Channel’s Width

In order to check whether the width of the central, lateral, and interanode channels has an impact on bubble evacuation, a case with these widths increased by 20 mm was simulated. The hypothesis was that increasing these widths would have the effect of reducing the BMI deformation.

#### 4.4.1 The Preferential Path of Bubbles

Table 9 shows the effect of increasing channel width on the bubbles’ preferential path.

**Table 9:**
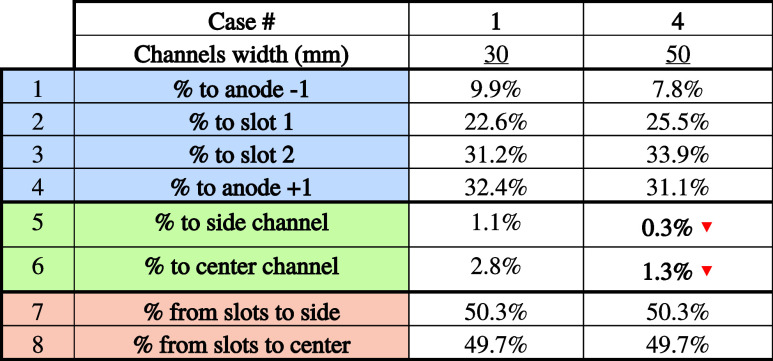
Path of Bubbles as a Function of Channel Width

In the table, the red and green arrows show variations greater than 20% compared with the base case.

There is no significant difference between the two cases studied in terms of the bubbles’ preferential path. This result goes in the same direction as expected since the two main forces acting on the bubbles are gravity and the flow of fluids due to MHD.

#### 4.4.2 The Tension

Figure 18 presents the effect of increasing the channel’s width on the tension.

**Figure 18:**
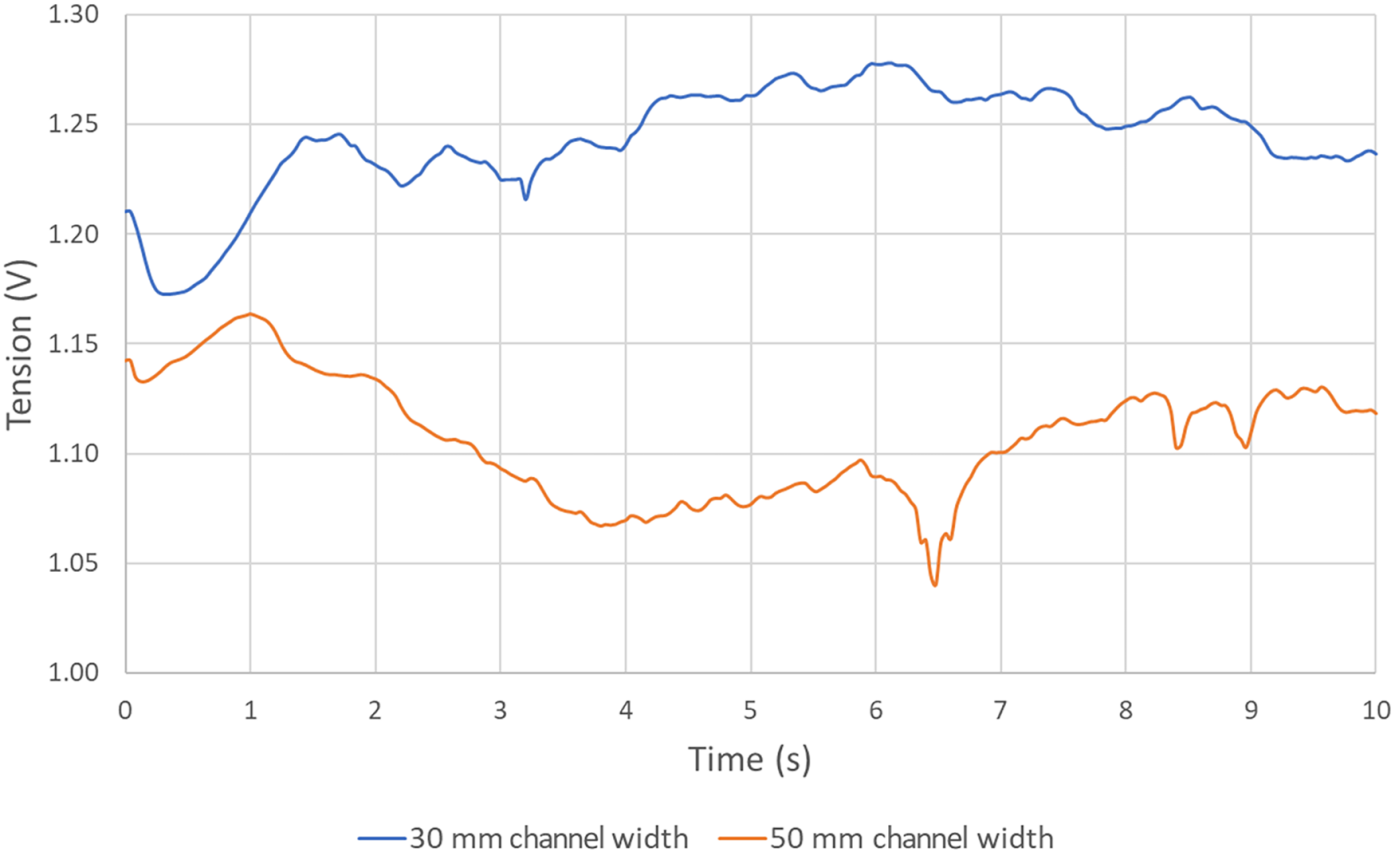
Effect of channel width on the mean tension.

As seen in Figure 18, in this particular case, increasing the width of the central, lateral, and interanode channels has a considerable effect on voltage since increasing this width by 66% reduces voltage by 12.6%. In fact, the average voltage in the case with 30 mm side channels is 1.26 V, whereas it is 1.10 V when the side channels are enlarged to 50 mm. This decrease in voltage is due to two main factors.

First, by increasing the available cathode area, resistance is reduced since it is inversely proportional to the surface. The changes in dimensions give an increase in total surface area in the xz plane of 8%, which represents about 0.1 V of the 0.15 V decrease.

Then, the hypothesis that explains the remaining difference is the random nature of the process. In fact, in the case with increased channel width, an important amount of gas seems to evacuate quickly compared to the base case. As a result of this early evacuation, the average tension is slightly lower than it would have been if bubbles had followed a behavior more similar to the “Base case”.

#### 4.4.3 The Bath–Metal Interface Deformation

In this model, part of the metal movement is caused by the escape of bubbles. As the bubbles escape from the bottom of the anode, they have the effect of creating movement in the electrolytic bath and in liquid aluminum. In this context, it is interesting to observe whether the width of the channels has an effect on the fluid movement when the bubbles escape.

Figure 19 shows that, contrary to expectations, the channel width has a very limited impact on the deformation of the bath–metal interface. A small variation in the median of the order of 0.1 mm is observed, which is not considered relevant in this context.

**Figure 19:**
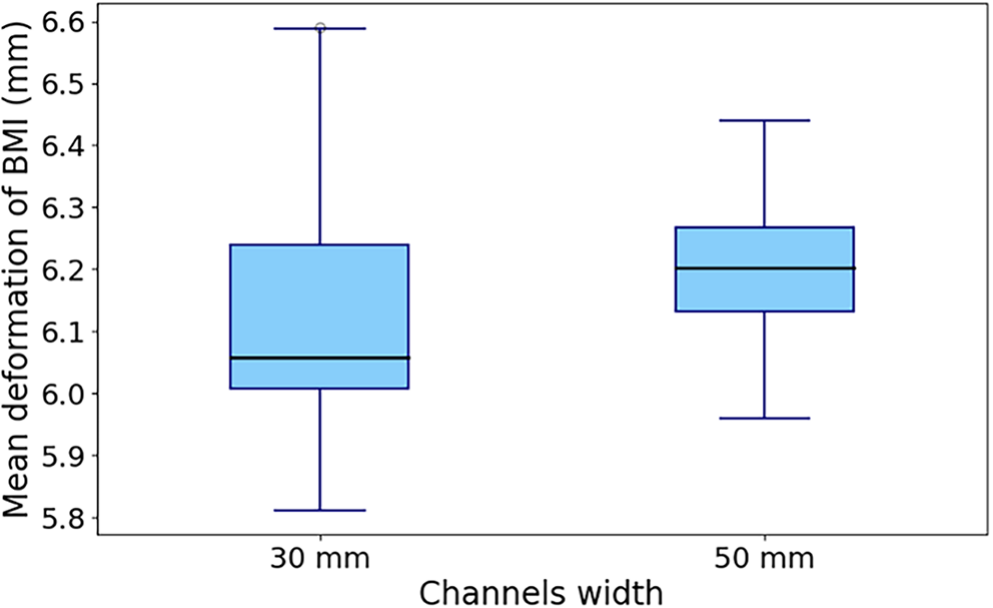
Effect of channel width on the mean BMI deformation.

#### 4.4.4 The Turbulent Kinetic Energy

In the central channel, the main objective is to maximize the alumina dissolution. To achieve this, a compromise has to be made between stirring and the volume of the electrolyte bath available. Figure 20 shows the turbulent kinetic energy in the center channel and in the side channel as a function of the channel width.

**Figure 20:**
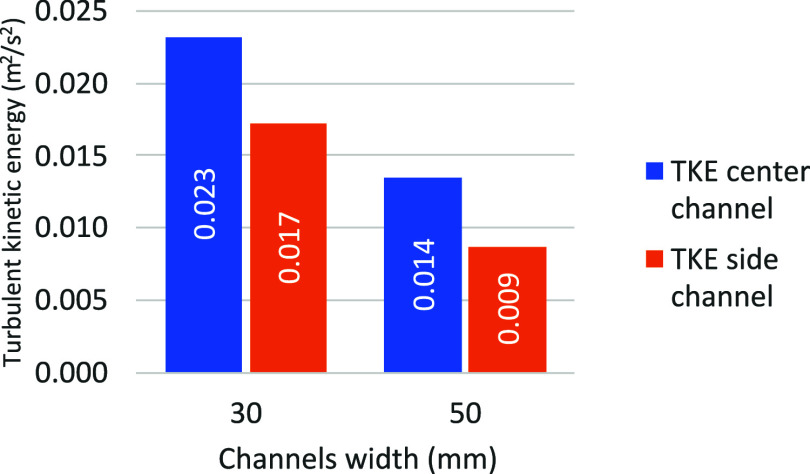
Effect of channel width on the mean TKE.

The simulations show that for an increase in the width of the central channel from 30 to 50 mm (66%), the average TKE decreases inversely proportionally (−41.6%). In other words, in both cases, the total turbulent kinetic energy remains more or less constant. In the case of the 30 mm central channel, this energy is more concentrated, while for the 50 mm central channel, more bath volume is available for the dissolution.

Similarly, the width of the side channel must be carefully controlled to optimize the available space while limiting the impact of bubbles on the wall to prevent wall erosion. Figure 20 also shows that for the side channel, the mean TKE decreased even more significantly with the increase in channel width. This result highlights the importance of the side channel’s width on the erosion caused by bubbles on the side wall. By going from 30 to 50 mm, the energy concentration is almost halved, therefore promoting the formation of side ledge.

### 4.5 Bubble Escape Frequency Analysis

#### 4.5.1 The Effect of Bubble Escape on Tension Fluctuations

In order to better understand the main escape frequencies of the bubbles under the anode, a fast Fourier transform (FFT) was performed on the voltage fluctuation resulting from the model where seconds #2 to #10 are selected for this analysis. In fact, to obtain a frequency analysis as meaningful as possible, the time period analyzed must be maximized. In these cases, the voltage signal stabilizes after about 2 s of simulation. Figure 21 presents the evolution of tension in time for case #1 as a reference.

**Figure 21:**
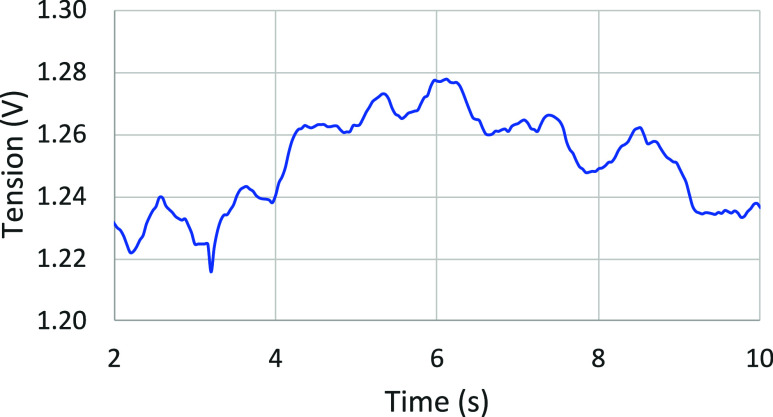
Voltage as a function of time (between 2 and 10 s of the simulation) for case #1.

Figure 21 shows various phenomena. Initially, there is a gradual increase in voltage over 3 s, followed by a decrease of about 3 s, corresponding respectively to gas accumulation and release. Simultaneously, some bubbles formed and released at higher frequencies. In particular, medium-sized bubbles can be detected between 0.5 and 2 Hz, and smaller variations can be seen several times per second.

Figure 22 presents the fast Fourier transform of all cases, which allows the analysis of the escape frequency of bubbles.

**Figure 22:**
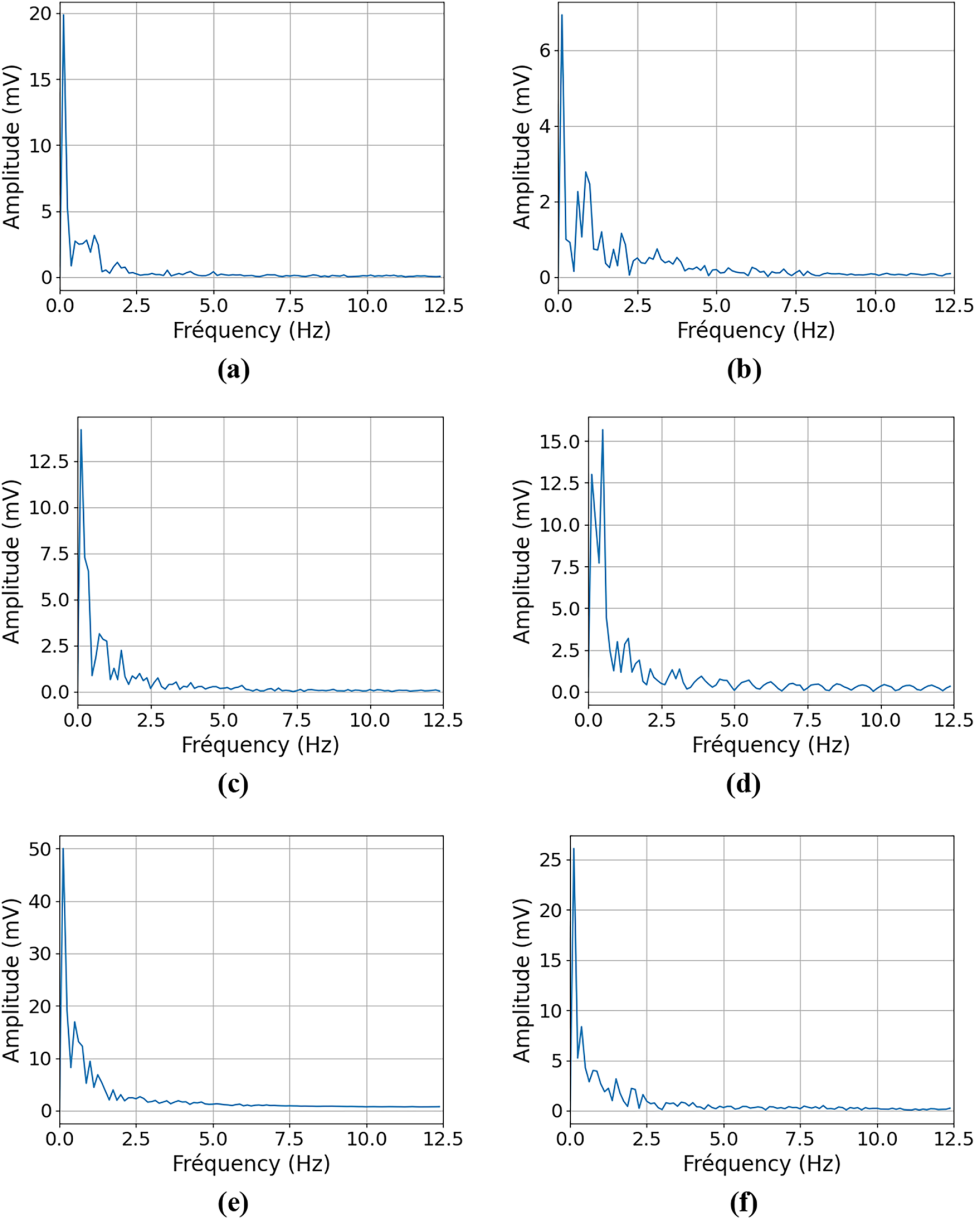
Fast Fourier transform (between 2 and 10 s of the simulation). (a) Base case (ACD = 30 mm). (b) 35 mm ACD. (c) 40 mm ACD. (d) MHD from side wall to center. (e) MHD from center to side wall. (f) 50 mm side channels width.

The figure shows an important fluctuation in tension caused by the gas that escapes at low frequencies under 0.5 Hz. It corresponds to the larger bubbles that accumulate near the anode ends and then escape when there’s enough accumulation. They have a minimum amplitude of around 5 mV and a maximum amplitude that can reach as much as 50 mV in some cases. Since 8 s are kept for each case, the smallest frequency that can be analyzed is 0.125 Hz. So, it is possible that other important frequencies are present under 0.125 Hz but are not covered in this work.

Medium-sized bubbles are evacuated at frequencies between 0.5 and 2 Hz. Depending on the case, their voltage variation is about four times smaller than that of large bubbles.

Then, many smaller bubbles are evacuated at frequencies of over 2 Hz. Their amplitude is generally under 1 mV as presented in Figure 23.

**Figure 23:**
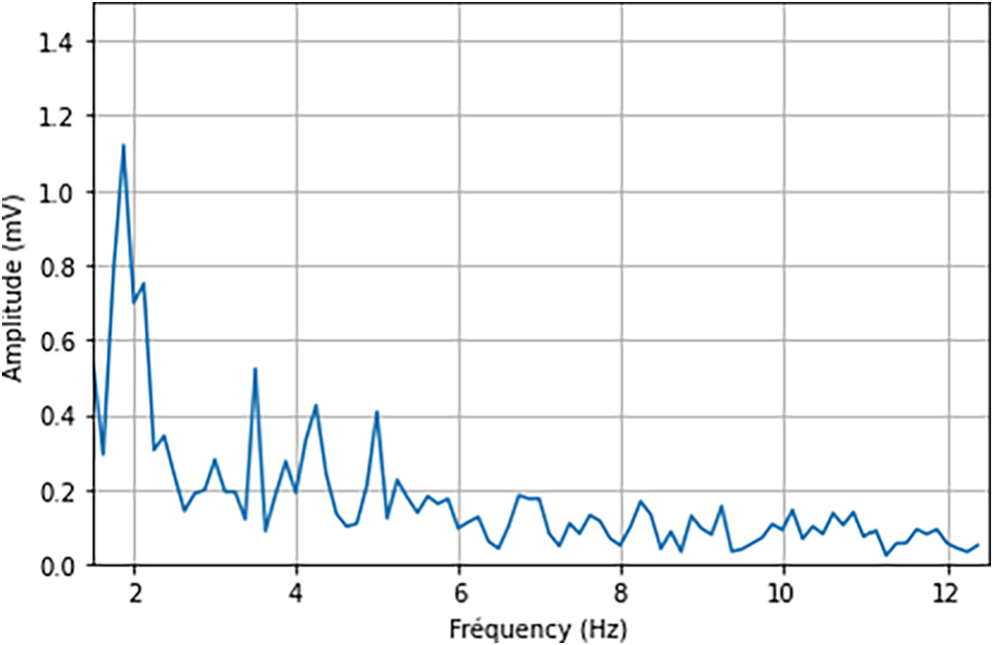
Fast Fourier transform of case 1 (zoom on frequencies 1.5 to 12.5 Hz).

Poncsák and Kiss^15,16^ studied bubble production, coalescence, and escape in detail using a numerical model. In their work, the simulated anodes were smaller, which would theoretically be favorable to facilitate the release of the bubble, thus reducing the average bubble size in comparison to this simulation. However, the anodes considered had no chamfers or anode tilt angles, which would, oppositely, reduce the escape frequency and enhance the size of the bubble.

In their work, they found a main bubble escape frequency slightly below 1 Hz, which is on a very similar order of magnitude as in this work. More importantly, they found that the frequency of escape decreased when the flow occurred along the length of the anode rather than along its width. Since bubbles have a longer way to go before escaping, gas accumulation and hence coverage rate is greater. These results are consistent with the observations from this work.

#### 4.5.2 Comparison of Largest Gas Accumulation between Cases

One way to compare cases between them is with the amplitude of the largest bubbles. Indeed, a larger amplitude indicates a greater accumulation of gas under the anode. A smaller bubble accumulation is advantageous for the cell since the tension imposed by the bubble resistance is lower.

Tables 10, 11, and 12 present respectively the effect of ACD, MHD direction, and channel width on the sum of amplitude of the large bubbles (frequencies lower than 0.5 Hz).

**Table 10:** Voltage Fluctuation of Largest Bubble as a Function of ACD

**case #**	**ACD [mm]**	**amplitude [mV]**
1	30	28.7
2	35	9.0
3	40	28.9

**Table 11:** Voltage Fluctuation of Largest Bubble as a Function of MHD Direction

**case #**	**MHD direction**	**amplitude [mV]**
1	from anode −1 to anode +1	28.7
4	from side wall to center	46.6
5	from center to side wall	94.2

**Table 12:** Voltage Fluctuation of Largest Bubble as a Function of Channel Width

**case #**	**channel width [mm]**	**amplitude [mV]**
1	30	28.7
6	50	40.9

The values presented in Tables 10, 11, and 12 give an important indicator of the accumulation of large bubbles. The most obvious effect can be seen in Table 11, specifically for case #5. Indeed, there is a flagrant increase in the accumulation of large bubbles, seeing the tension resulting from the coalescence of bubbles rise from 28.7 to 94.2 mV. This reflects the very significant accumulation of gas under the anode, as the MHD force acts against the effect of the anode tilt. A lesser but also noteworthy increase is seen in case #4, where the MHD acts on the anode length direction rather than the width direction. This additional gas accumulation occurs because the MHD-induced force acts to push the bubbles along the length of the anode rather than toward the slots, leading to a higher distance to move for the bubble before evacuating the anode.

Table 10 does not show a consistent pattern between the effect of ACD and the bubble, indicating that a variation of ± 20 mV is likely within the expected fluctuations range of the simulation. Consequently, it is impossible to confirm if the variation from Table 12 is significant as they show a similar variation of the amplitude, which may be exclusively attributed to the randomness of the fluid flow.

## 5 Validation

In order to validate the results from the simulations presented in the previous section, voltage drop measurements were taken in an industrial environment. A Hioki lr8431-20 data logger was used to acquire data on 10 anodes simultaneously at a frequency of 100 Hz. One hour of data was acquired on these 10 anodes. For this measurement, voltage probes were planted in the anode stems at a distance of 20 cm from each other. Anode assemblies 1 to 5 and 16 to 20 were instrumented. Figure 24 shows a schematic of the cell with the position of the anodes. Anodes 1 to 10 are on the upstream side of the pot.

**Figure 24:**
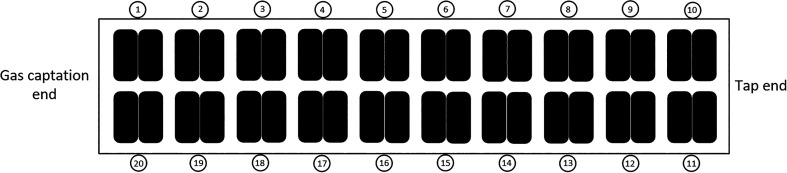
Position of the anodes in the pot.

Figure 25 shows the evolution of the voltage drop measured for the anodes. For ease of reading, only 10 s are shown.

**Figure 25:**
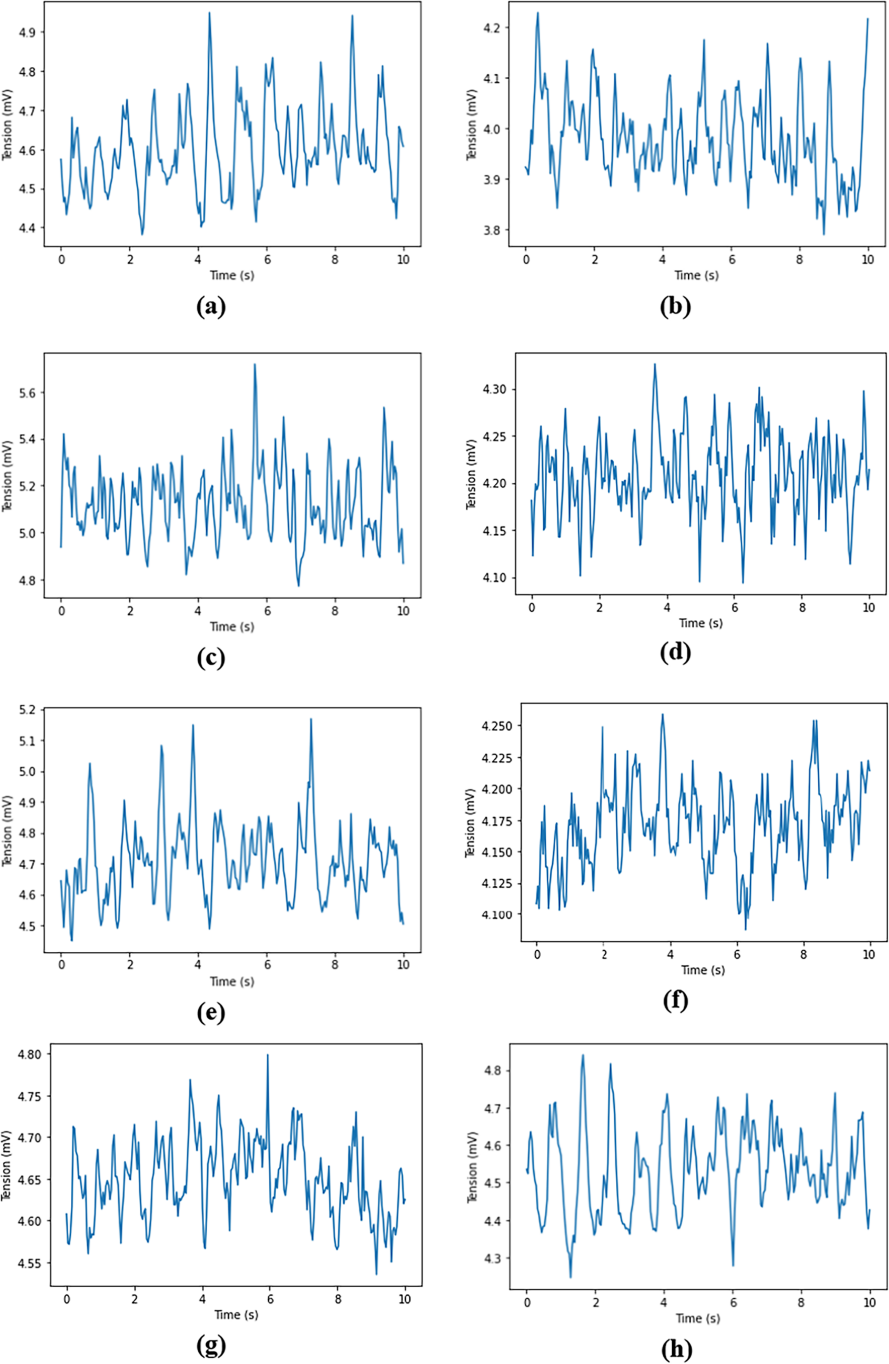

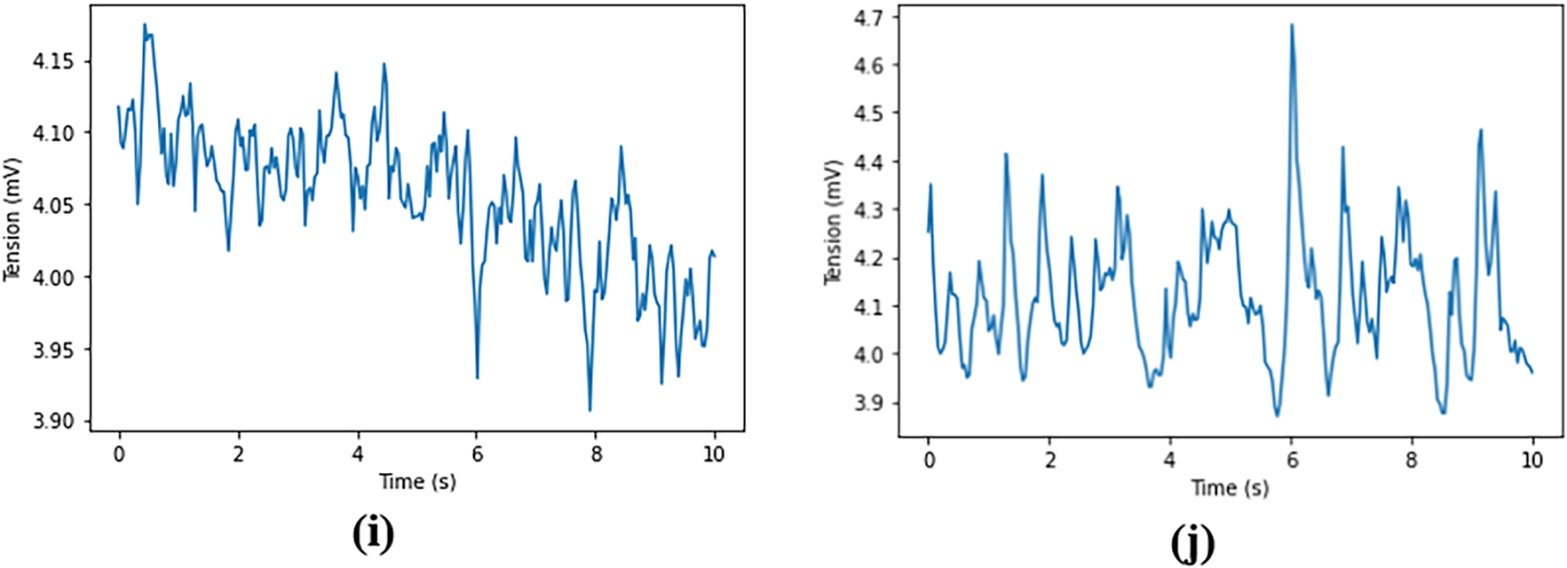
Evolution of tension drop for anode 1 over 10 s. (a) Anode 1. (b) Anode 2. (c) Anode 3. (d) Anode 4. (e) Anode 5. (f) Anode 16. (g) Anode 17. (h) Anode 18. (i) Anode 19. (j) Anode 20.

Differences can be observed between the experimental values presented in Figure 25 and the voltage values obtained from the simulations in Figure 21.

First, the voltage values are not of the same order of magnitude simply because the voltage given in the simulations corresponds to the voltage drop between the anode and cathode, whereas in the experimental data, it corresponds rather to the voltage drop across 20 cm of the anode stem. In the simulation, the current (I) is constant; therefore, the tension (V) is only modified as the effect of the resistance (R), which can change only due to the presence of bubbles. In the real cell, the tension drop in the stem is measured, while its resistance is constant. Thus, by measuring the tension, the effect on the amperage that passes in the anode is measured. The hypothesis is therefore that for these tests and for the frequency analyzed (0.1 to 15 Hz), the amperage fluctuation in the anode stem will be directly a result of the gas layer’s extra resistance.

Second, there seem to be faster variations in the voltage of experimental data than in the simulation data. Experimentally measured data on an anode of a cell in operation are greatly influenced by the presence of bubbles not only on this anode but also on the 19 others. In addition, the complex MHD in the cell and the effect of varying liquid aluminum heights throughout the cell also have an effect on the measured voltage. Also, for the numerical model, only one anode block is represented, where for the experimental data, it is an anodic assembly (2 anode blocks). Looking at Figure 25, the most important fluctuation present is around 1 Hz. Figure 26 shows the FFTs of the experimental data for the 10 anodes.

**Figure 26:**
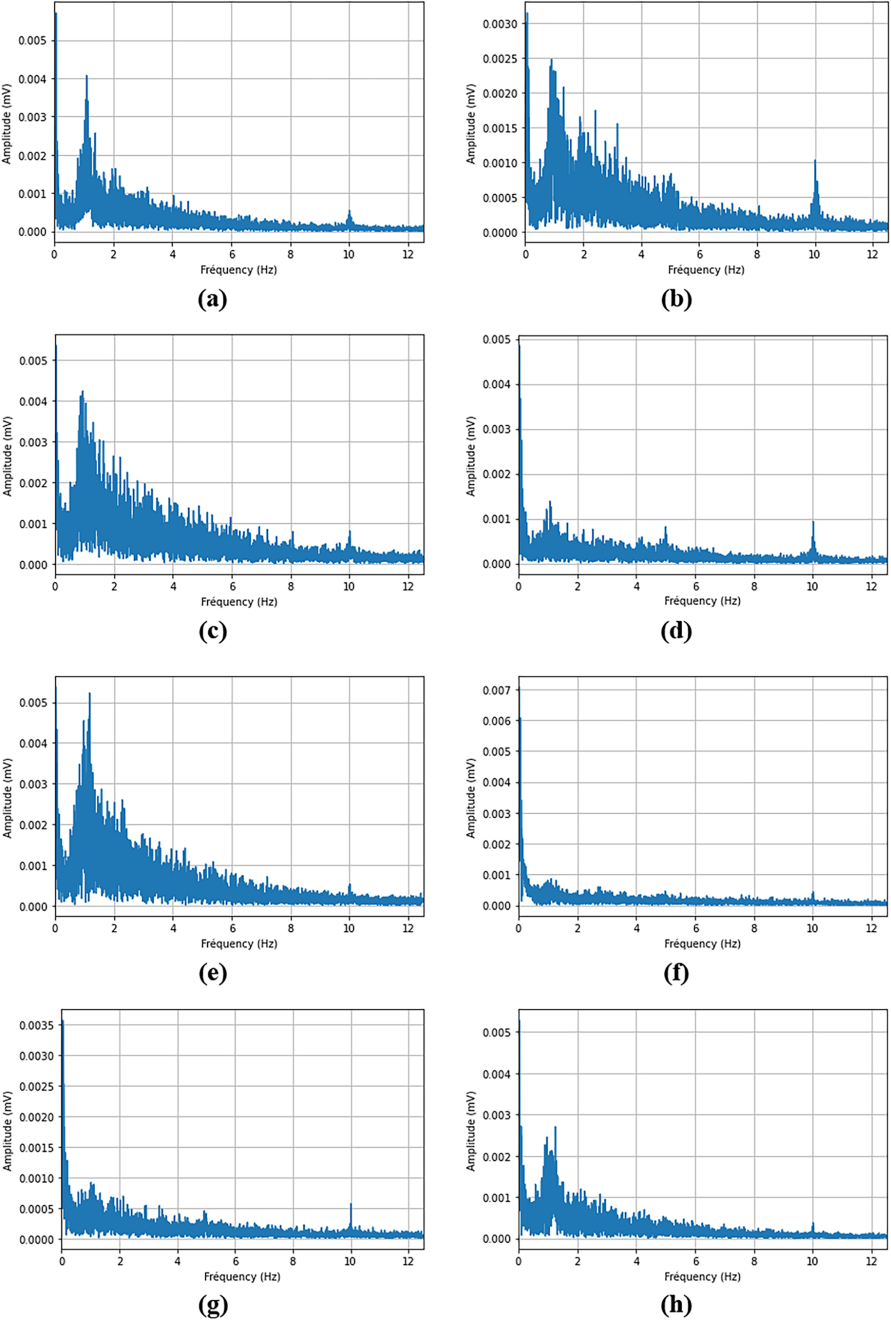

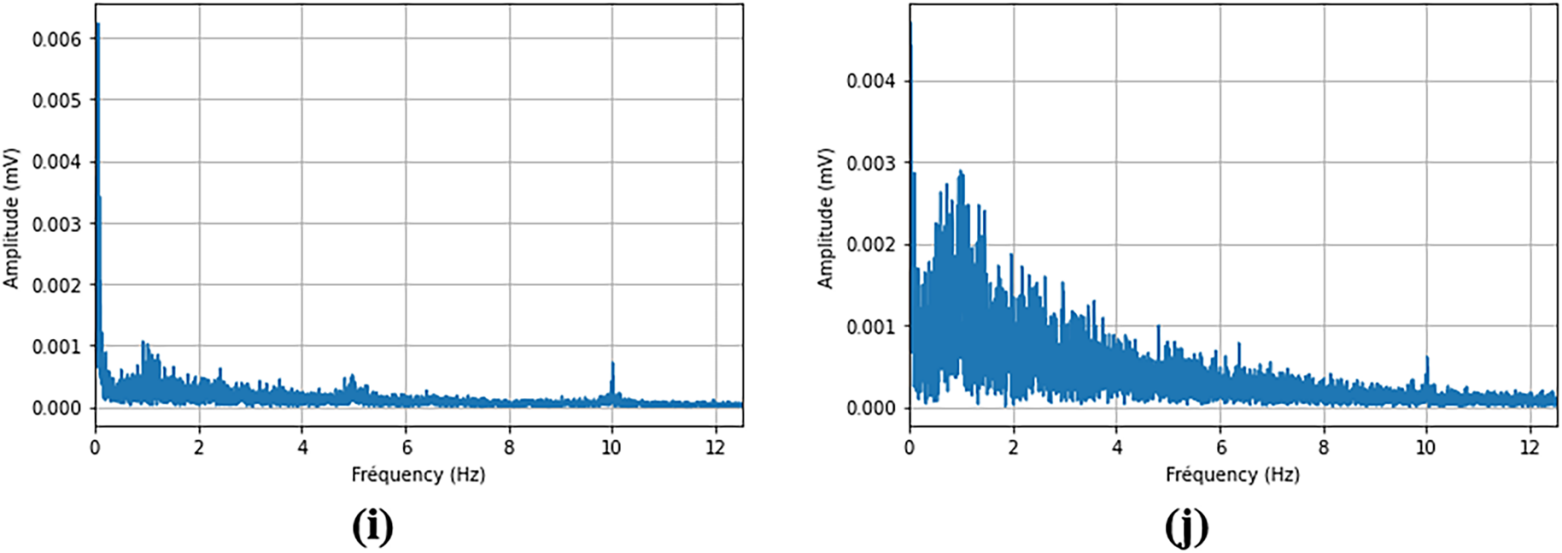
FFT analysis of the voltage drop for the 10 anodes measured. (a) Anode 1. (b) Anode 2. (c) Anode 3. (d) Anode 4. (e) Anode 5. (f) Anode 16. (g) Anode 17. (h) Anode 18. (i) Anode 19. (j) Anode 20.

Table 13 gives the age of each anode.

**Table 13:** Age of the Anodes

**anode**	**age (days)**
1	25
2	9
3	23
4	4
5	18
16	11
17	1
18	19
19	14
20	6

The first observation is that there seems to be an important frequency around 1 Hz. In fact, this very significant oscillation is observed around the same frequency for all of the anodes studied. This result differs slightly from that observed in the numerical simulations. In the simulations, a phenomenon was also seen to be a little under 1 Hz, but it was less pronounced. Instead, there was more of a build-up over a cycle of around 10 s, but it is not observed on every anode in the experimental data. However, some anodes still show a significant build-up over cycles of around 5 to 10 s, but with a lower amplitude as shown in Figure 25. In particular, these oscillation cycles are best seen on anodes 16 to 19 (Figure 25f–i). As a result, the evacuation cycles of these anodes and their FFT analyses are more similar to those given by the numerical model.

Then, it can be seen that for each anode, significant frequencies are also observed at lower frequencies (<0.1 Hz), which on average have amplitudes around twice as great as the frequency at 1 Hz (varies according to the anode). However, these results cannot be compared with the simulations that were carried out, since the smaller frequency that can be analyzed is 0.125 Hz. On the other hand, the assumption made here is that these frequencies are not due to bubble evacuation, but rather to the feeding cycles and macro-movement of the metal pad. More detailed analyses will be needed to clarify these results.

Finally, a spike of non-negligible amplitude is present at 10 Hz on most FFTs, but particularly on certain anodes such as anodes 2, 4, and 17 (Figure 26b,d,g). It is interesting to note in Table 13 that these are the three newest anodes. It suggests there could be a correlation between the age of the anode and the intensity of that spike. However, the nature of this peak remains uncertain, but it could potentially correspond to A frequency of evacuation of small bubbles through the slots, a resistance fluctuation of neighboring anodes, a parasitic signal coming from the measuring device used for the measurements, or a signal coming from the instrumentation on the cell.

## 6 Conclusions

The model enabled a better understanding of the effect of different parameters on the cell response.

In particular, simulations have shown that BMI deformation appears to decrease with increasing ACD. The simulations also showed a near-linear increase in the voltage drop as a result of the ACD change due to the electrolytic bath resistance, consistent with the expected behavior. Investigations regarding the mixing of electrolyte, using turbulent kinetic energy in the center and side channels, revealed that the permanent anode angle (resulting from permanent metal pad deformation) is a significant factor influencing the mixing of the electrolyte in region beneficial for the electrolysis cells.

Various important observations were then made in relation to the flow direction of the MHD. First, it was found that the direction of metal flow has a major impact on bubble evacuation paths. The change in the bubbles’ preferential flow path was particularly noticeable in the average voltage, which increased by 90 mV when the MHD flow changed by 90° underneath the anode surface. Consequently, the results confirm that the MHD flow plays a significant role in the uneven conditions of the cell by changing the local bubble overvoltage and changing the alumina dissolution conditions and heat transfer coefficient with the ledge. By correlating these results with the MHD flow pattern of the cell, it is possible to pinpoint the regions of the pot that are most affected, positively or negatively, by these phenomena.

The study then showed the effect of the width of the central, lateral, and interanode channels on voltage drop. By increasing these widths from 30 to 50 mm, the voltage dropped by 150 mV since the current circulates with less resistance.

The frequency analysis highlighted the most significant escape frequency of large bubbles between 0.125 and 0.5 Hz. These bubbles create the greatest voltage variations, up to 50 mV.

Finally, experimental values were acquired to validate the results. The data showed that the main frequencies observed in the simulations were also present in the actual pot. However, the analysis of gas evacuation indicates that bubbles escape a little more easily in the industrial pot than in the model, notably due to the many complex behaviors specific to the cell, which were neglected in the model. These data then show different behaviors between the anodes, possibly due to the movement of the MHD locally under the anode as well as to its age, which will affect the curvature of the anode following different states of oxidation.

Ultimately, this work strongly highlights the non-negligible influence of bubble flow behavior, which significantly plays a role in the heterogeneity of electrolysis cells by affecting the electrical, thermal, and chemical dynamics of the whole process.
